# Identification of CRF89_BF, a new member of an HIV-1 circulating BF intersubtype recombinant form family widely spread in South America

**DOI:** 10.1038/s41598-021-90023-x

**Published:** 2021-06-01

**Authors:** Elena Delgado, Aurora Fernández-García, Marcos Pérez-Losada, María Moreno-Lorenzo, Ismael Fernández-Miranda, Sonia Benito, Vanessa Montero, Horacio Gil, Silvia Hernáez, Josefa Muñoz, Miren Z. Zubero-Sulibarria, Elena García-Bodas, Mónica Sánchez, Jorge del Romero, Carmen Rodríguez, Luis Elorduy, Elena Bereciartua, Esther Culebras, Icíar Rodríguez-Avial, María Luisa Giménez-Alarcón, Carmen Martín-Salas, Carmen Gómez-González, José J. García-Irure, Gema Cenzual, Ana Martínez-Sapiña, María Maiques-Camarero, Lucía Pérez-Álvarez, Michael M. Thomson

**Affiliations:** 1 HIV Biology and Variability Unit, Centro Nacional de Microbiología, Instituto de Salud Carlos III, Majadahonda, Madrid, Spain; 2 Consortium for Biomedical Research in Epidemiology and Public Health (CIBERESP), Instituto de Salud Carlos III, Madrid, Spain; 3 Department of Biostatistics and Bioinformatics, Computational Biology Institute, Milken Institute School of Public Health, George Washington University, Washington, DC USA; 4 CIBIO-InBIO, Centro de Investigação em Biodiversidade e Recursos Genéticos, Universidade do Porto, Campus Agrário de Vairão, Vairão, Portugal; 5 Hospital Universitario Basurto, Bilbao, Spain; 6 Centro Sanitario Sandoval, Madrid, Spain; 7 Hospital Universitario Cruces, Instituto de Investigación Biocruces, Baracaldo, Vizcaya Spain; 8 Hospital Clínico Universitario San Carlos, Madrid, Spain; 9 Hospital Virgen de La Luz, Cuenca, Spain; 10 Complejo Hospitalario de Navarra, Pamplona, Spain; 11 Hospital Universitario Araba, Vitoria-Gasteiz, Spain; 12 Hospital Reina Sofía, Tudela, Navarra Spain; 13 Hospital Universitario Severo Ochoa, Leganés, Madrid, Spain; 14 Hospital Universitario Miguel Servet, Zaragoza, Spain; 15 Hospital Nuestra Señora del Prado, Talavera de la Reina, Toledo Spain; 16 Present Address: Instituto de Investigación Sanitaria Puerta de Hierro-Segovia de Arana, Majadahonda, Madrid, Spain

**Keywords:** Retrovirus, Viral evolution

## Abstract

Circulating recombinant forms (CRFs) contribute substantially to the HIV-1 pandemic. Among 105 CRFs described in the literature, 16 are BF intersubtype recombinants, most of South American origin, of which CRF12_BF is the most widely spread. A BF recombinant cluster identified in Bolivia was suggested to represent a new CRF_BF. Here we find that it belongs to a larger cluster incorporating 39 viruses collected in 7 countries from 3 continents, 22 of them in Spain, most from Bolivian or Peruvian individuals, and 12 in South America (Bolivia, Argentina, and Peru). This BF cluster comprises three major subclusters, two associated with Bolivian and one with Peruvian individuals. Near full-length genome sequence analyses of nine viruses, collected in Spain, Bolivia, and Peru, revealed coincident BF mosaic structures, with 13 breakpoints, 6 and 7 of which coincided with CRF12_BF and CRF17_BF, respectively. In a phylogenetic tree, they grouped in a clade closely related to these CRFs, and more distantly to CRF38_BF and CRF44_BF, all circulating in South America. These results allowed to identify a new HIV-1 CRF, designated CRF89_BF. Through phylodynamic analyses, CRF89_BF emergence was estimated in Bolivia around 1986. CRF89_BF is the fifth CRF member of the HIV-1 recombinant family related to CRF12_BF.

## Introduction

One of the distinguishing features of HIV-1 evolution is its high recombination rate, which can be similar to or even greater than its mutation rate^1,2^. HIV-1 features promoting recombination include large viral population sizes, rapid viral turnover^3^, frequent multiply-infected cells in lymphoid organs^4^, and high genetic diversity, which facilitates superinfection with genetically divergent variants, since susceptibility to interclade immune responses correlates with genetic distance separating the variants eliciting them^5^. Recombination in HIV-1 can increase viral diversity^2,6^, augment replicative fitness^7–9^, promote evasion from immune responses^6,10,11^, and facilitate propagation of drug resistance mutations^12^.

Recombination has contributed extensively to the generation of genetic diversity in the HIV-1 pandemic^13,14^. Recombinant forms are generated in individuals infected with two or more HIV-1 clades. Those found in a single individual or a single epidemiologically-linked cluster are designated unique recombinant forms (URFs) and those found in three or more epidemiologically-unlinked individuals are designated circulating recombinant forms (CRFs)^15^, of which 105 have been described in the literature. The proportion of CRFs has increased over time in the HIV-1 pandemic, representing around 17% infections in 2010–2015^14^. Among identified CRFs, the most numerous are those derived from parental strains of subtype B and subsubtype F1, of which 16 have been reported, most of them originated in South America. The first reported CRF_BF was CRF12_BF^16–18^, which circulates widely in Argentina and Uruguay^16–23^ and in lower proportions in other countries from South America^24–26^. Subsequently, three other CRFs related to CRF12_BF (CRF17_BF^27^, CRF38_BF^23^, and CRF44_BF^28^) were identified in different South American countries, mainly in the South Cone. Numerous URFs closely related to CRF12_BF, as shown by coincident breakpoints and grouping in phylogenetic trees, have also been identified in some of these countries^17,18,20,29,30^. It has been proposed that all these recombinants constitute a “family”^31,32^ of viruses that derive from a common recombinant ancestor, probably generated in Brazil from locally circulating B and F strains; subsequently, this ancestor would have gone through successive rounds of recombination with subtype B viruses, generating a great diversity of recombinant forms, some of which propagated epidemically, becoming CRFs^29^. Here we identify a fifth CRF member of the CRF12_BF-related family.

## Materials and methods

Samples from HIV-1-infected individuals were collected in 14 Spanish regions for a molecular epidemiological study. An ~ 1.4 kb pol fragment in protease-reverse transcriptase (Pr-RT) was amplified by RT-PCR/nested PCR from plasma RNA as described previously^33^ and sequenced with the Sanger method using a capillary automated sequencer. Near full-length genome (NFLG) sequences were obtained for selected samples by amplification in four overlapping segments from plasma RNA and sequenced by the Sanger method, as described^29,34^. Newly derived sequences are deposited in GenBank under accessions KX818199, KX818200, MW344906-MW344922, and MW802822-MW802825 (Table 1).

**Table 1:** Epidemiological data of patients residing in Spain studied by us and GenBank accessions of sequences.

Sample ID	City of sample collection	Region of sample collection	Year of sample collection	Year of HIV diagnosis	Gender	Transmission route	Country of origin	GenBank accession (Pr-RT)	GenBank accession (NFLG)
CU0019	Cuenca	Castilla-La Mancha	2016	2015	F	Heterosexual	Spain	MW344905	
CU0020	Cuenca	Castilla-La Mancha	2016	2009	M	Heterosexual	Spain	MW344906	
M0849	Madrid	Madrid	2016	2016	M	Heterosexual	Bolivia	MW344907	
M1063	Madrid	Madrid	2017	2017	F	Heterosexual	Peru	MW344908	
M1079	Madrid	Madrid	2017	2017	M	Heterosexual	Peru	MW344909	MW802822
M1131	Madrid	Madrid	2017	2017	M	MSM	Peru	MW344910	
MS0254	Madrid	Madrid	2018	2018	M	MSM	Peru	MW344911	
MS0360	Madrid	Madrid	2019	2019	M	MSM	Peru	MW344912	MW802823
NA0239	Tudela	Navarra	2016	2016	M	Heterosexual	Peru	MW344913	
NA0379	Pamplona	Navarra	2018	2018	F	Heterosexual	Bolivia	MW344914	
P2345	Bilbao	Basque Country	2009	2009	F	Heterosexual	Bolivia	MW344915	
P2346	Bilbao	Basque Country	2009	2009	M	Sexual	Spain	MW344916	
P2633	Bilbao	Basque Country	2010	2010	F	Heterosexual	Bolivia	MW344917	KX818199
P3174	Bilbao	Basque Country	2012	2012	M	Heterosexual	Spain	MW344918	
P3177	Bilbao	Basque Country	2012	2012	F	Heterosexual	Bolivia	MW344919	KX818200
P4464	Bilbao	Basque Country	2016	2015	M	MSM	Spain		MW344920
P5090	Vitoria	Basque Country	2018	2018	M	Sexual	Bolivia	MW344921	
TO0275	Toledo	Castilla-La Mancha	2020	2020	M	MSM	Spain		MW802824
Z0275	Zaragoza	Aragon	2018	2018	M	n.a	Bolivia	MW344922	MW802825

*n.a.* not available.

Sequences were aligned with MAFFT v7^35^. Initial phylogenetic trees with all Pr-RT sequences obtained by us were constructed via approximate maximum likelihood in FastTree2^36^, using the general time reversible evolutionary model with CAT approximation for among-site rate heterogeneity and assessment of node support with Shimodaira-Hasegawa (SH)-like local support values^37^. Subsequent maximum likelihood (ML) trees with sequences of interest were constructed in IQ-Tree^38^, using the best-fit substitution model determined by the program^39^, with assessment of node support with the ultrafast bootstrap approximation approach^40^. Trees were visualized with MEGA v7.0^41^. A phylogenetic network of NFLG sequences was also constructed with SplitsTree4^42^. In this analysis, the HKY + G + I evolutionary model was used (GTR is not available) and a 95% confidence network was estimated.

Mosaic structures were analyzed by bootscanning^43^ with SimPlot v1.3.5^44^, with tree construction using the neighbor-joining method and a window width of 250 nucleotides. Recombinant segments identified with SimPlot were further phylogenetically analyzed through ML with IQ-Tree and PhyML v3.0^45^ (with assessment of node support in the PhyML analyses with the approximate likelihood ratio test, Shimodaira Hasegawa-like (aLRT SH-like) procedure^37^) and through Bayesian inference with MrBayes v3.2^46^. The analysis with MrBayes was performed using the GTR + G + I substitution model. We ran two simultaneous independent runs and 8 chains 2–5 million generations long, ensuring that both runs reached convergence, as determined by an average standard deviation of split frequencies < 0.01. We discarded the first 50% of the trees in the posterior distribution as burn-in. The existence of adequate phylogenetic signal in short (< 200 nt) segments was analyzed through likelihood mapping^47^ with IQ-Tree.

Intersubtype breakpoint locations were also analyzed with GARD^48^, RDP4^49^ (with RDP, Geneconv, Chimaera, MaxChi, Bootscan, Siscan, and 3Seq methods implemented in it), and jpHMM^50^.

The time of emergence and most probable country location of the most recent common ancestor (MRCA) of the identified cluster and subclusters were estimated using Pr-RT sequences with the Bayesian Markov chain Monte Carlo (MCMC) coalescent method implemented in BEAST v1.8.4^51^. For this analysis, the positions in the alignment corresponding to codons containing antiretroviral drug resistance mutations in any of the sequences, as determined with Stanford University’s database HIVdb program^52^, were removed. Prior to the BEAST analysis, the existence of temporal signal in the dataset was analyzed with Tempest^53^. Since according to this analysis there was insufficient temporal signal, we used as a prior parameter a normally-distributed substitution rate (1.33 × 10^–3^ ± 2.57 × 10^–4^ subst./site/year) estimated from 65 CRF12_BF sequences, which exhibited an adequate temporal signal (r^2^ = 0.389 in TempEst analysis) (Supplementary Fig. S1). The BEAST analysis was performed using the SRD06 codon-based evolutionary model^54^, an uncorrelated lognormal relaxed clock model and the Bayesian Skyline Plot population growth model^55^. The MCMC was run for 20 million generations, ensuring that effective sample size values of all parameters were > 200, which indicates proper mixing. The posterior distribution of trees was summarized in a maximum clade credibility (MCC) tree with TreeAnnotator v1.8.4, after removal a 10% burn-in. MCC trees were visualized with FigTree v1.4.2 (Rambaut, http://tree.bio.ed.ac.uk/software/figtree/). Parameter uncertainty was summarized in 95% highest posterior density (HPD) intervals.

### Ethics declaration

This study was approved by the Research Ethics Committee of Instituto de Salud Carlos III, Madrid, Spain. Informed consent was obtained from all participants. All methods were performed in accordance with the relevant guidelines and regulations.

## Results

**Figure 1:**
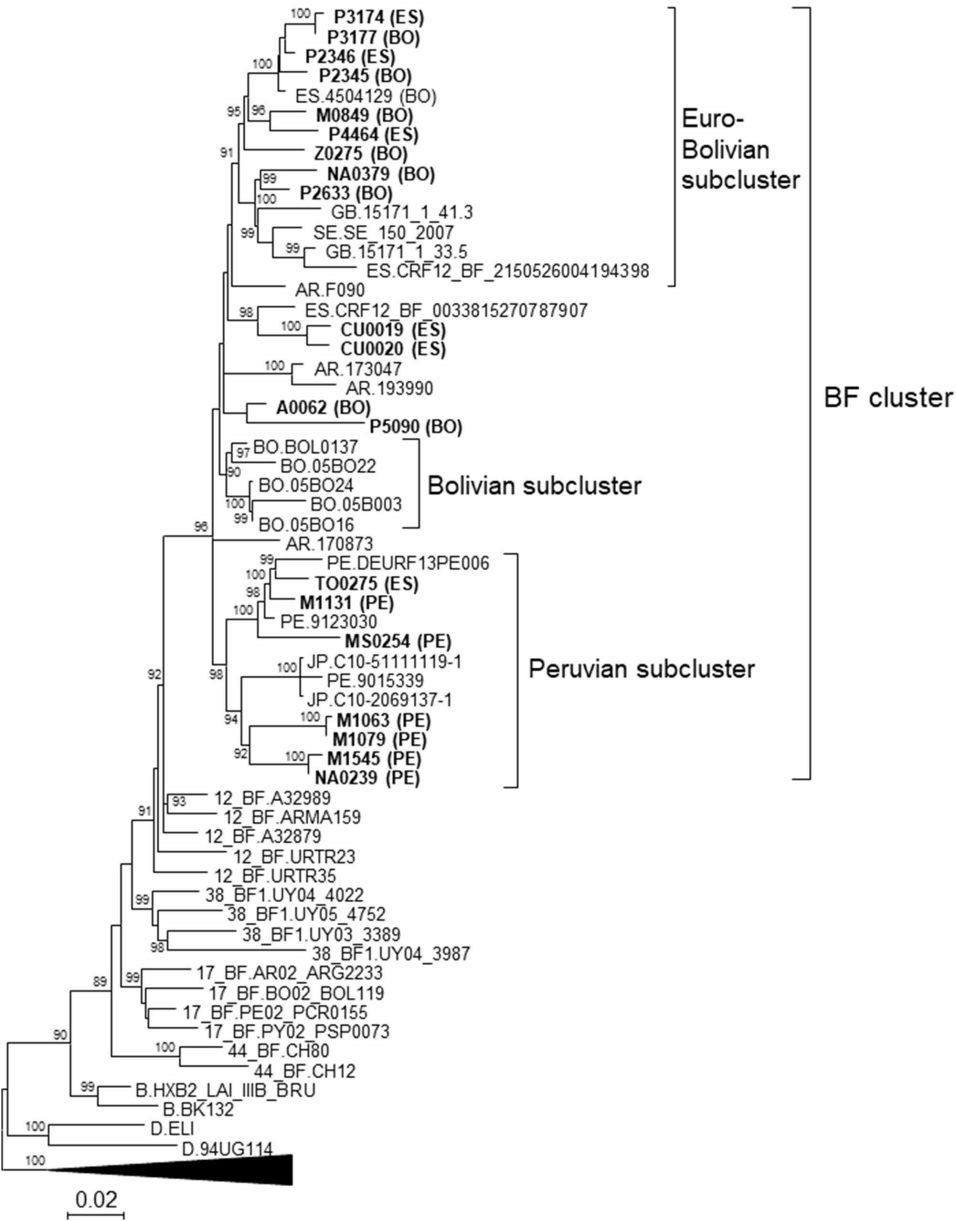
Maximum likelihood tree of Pr-RT sequences of BF cluster. Names of sequences obtained by us, all collected in Spain, are in bold type. Two-letter ISO code of country of origin of the individual, when known, is in parentheses after the virus name. In database sequences branching in the BF cluster, the country of sample collection is indicated before the virus name with the two-letter ISO country code. In reference sequences, subtype or CRF is indicated before the virus name. Branches corresponding to references of subtypes A, C, F, G, and H are compressed. Only bootstrap values ≥ 80% are shown.

In an HIV-1 molecular epidemiological study in Spain, we identified a phylogenetic cluster of 19 Pr-RT sequences from samples collected in five regions, nested within the CRF12_BF clade. In bootscan analyses, sequences from this cluster (henceforth, BF cluster) exhibited 5’-B/F/B/F-3’ recombinant structures that were very similar to each other (Supplementary Fig. S2a-f). Their structures also showed some similarity with that of CRF12_BF, from which they differed in a longer subtype B segment in the Pr-RT junction (Supplementary Fig. S2g-i). To determine whether additional sequences in databases clustered with viruses of the BF cluster, all BF recombinant Pr-RT sequences ≥ 900 nt long deposited in the Los Alamos HIV-1 sequence database^56^ and an additional BF recombinant sequence described in^57^, not available in the Los Alamos database but deposited in GenBank^58^ (accession MF109665), were downloaded and phylogenetically analyzed with FastTree2. We found that 20 additional database sequences fell in the BF cluster, which was also well supported in a ML tree constructed with IQ-Tree (Fig. 1). Of the 39 viruses belonging to the BF cluster, 22 were collected in Spain, 5 in Bolivia, 4 in Argentina, 3 in Peru, 2 in the United Kingdom, 2 in Japan, and 1 in Sweden. Epidemiological data from samples collected in Spain, available for all samples processed by us (Table 1) and from one database sequence, indicated that individuals in the BF cluster residing in Spain were predominantly male (except for 6 out of 19), of South American origin (from Bolivia or Peru, but 5 were native Spanish), and infected via heterosexual contact (but 5 of 18 were MSM). Transmission route information was also available from one sample collected in Peru (DEURF13PE006), which was from a MSM. Available clinical data for the samples collected in Spain and studied by us are shown in Supplementary Table 1.

In the BF cluster there were three well supported subclusters. One comprised 14 sequences, all collected in Western Europe, mostly in Spain, but also 2 in the UK and 1 in Sweden; for 10 of the 11 viruses collected in Spain, the country of origin of the patient was known, which was Bolivia in 8 and Spain in 2. A second subcluster comprised 5 viruses collected in La Paz, Bolivia. The third subcluster comprised 12 sequences from samples collected in Spain (n = 7), Peru (n = 3), and Japan (n = 2), with all but one samples from Spain being from Peruvian individuals. These sublcusters were designated Euro-Bolivian, Bolivian, and Peruvian, respectively (Fig. 1). Interestingly, 4 out of 5 viruses (M1131, MS0254, TO0275, and DEURF13PE006) grouping in a sub-subcluster within the Peruvian subcluster were from MSM.

**Figure 2:**
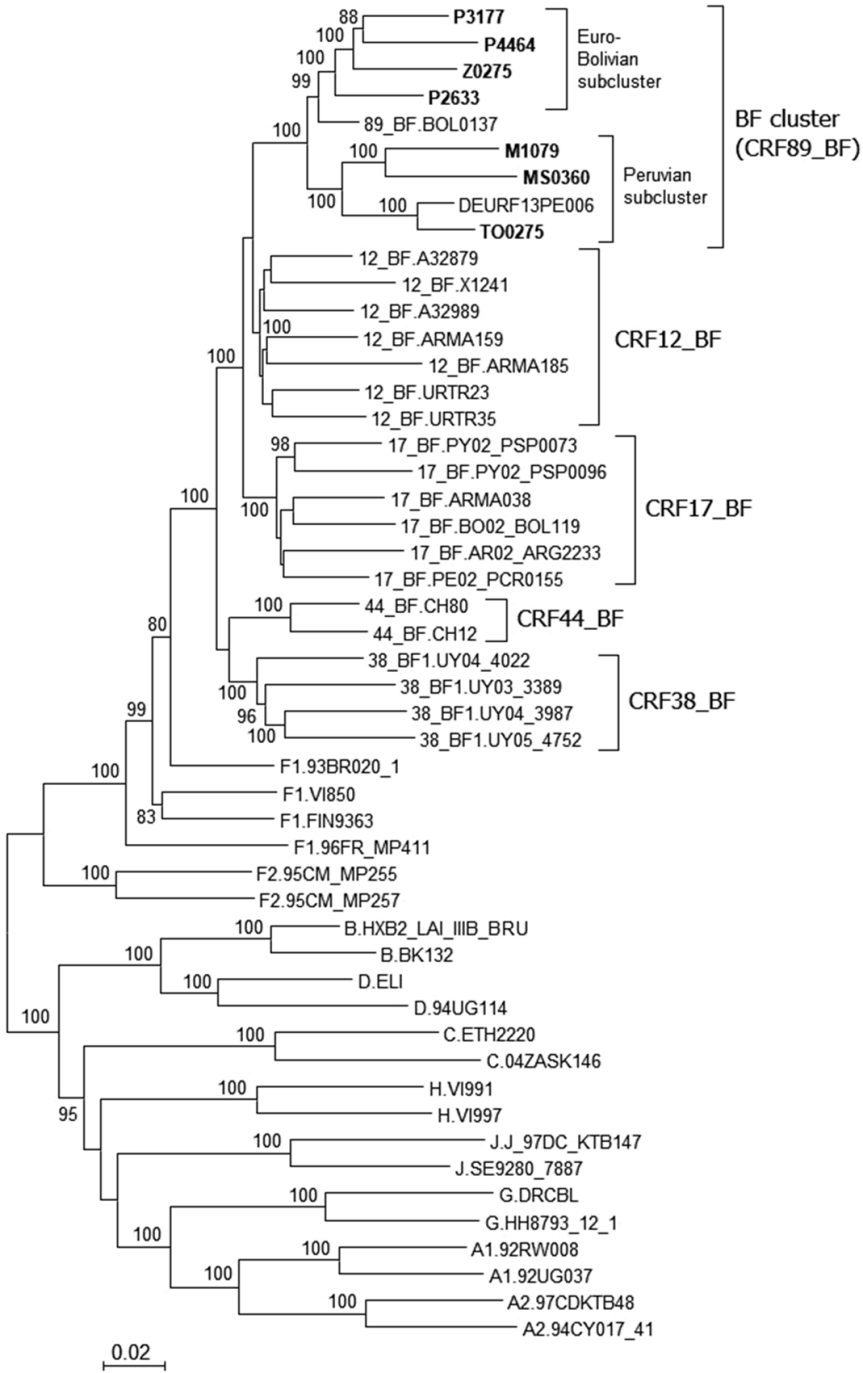
Maximum likelihood tree of NFLG sequences of the BF cluster and of references of CRF_BFs from the Southern Cone of South America and of subtypes. Names of sequences obtained by us are in bold type. In database sequences located in the BF cluster, the country of sample collection is indicated before the virus name with the two-letter ISO country code. In reference sequences, the subtype or CRF is indicated before the virus name. Only bootstrap values ≥ 80% are shown.

We obtained NFLG sequences from seven viruses in the BF cluster: three from samples collected in the city of Bilbao from individuals without known epidemiological links, two (P2633 and P3177) from Bolivian individuals and one (P4464) from a Spanish individual; two (M1079 and MS0360) from samples from Madrid, both from Peruvian individuals; one (Z0275) from Zaragoza, from a Bolivian individual; and one (TO0275) from Toledo, from a Spanish individual. In a phylogenetic tree that included two other database NFLG sequences of viruses of the BF cluster collected in Bolivia (BOL0137)^18^ and Peru (DEURF13PE006), respectively, the viruses of the BF cluster grouped in a strongly supported clade (100% ultrafast bootstrap support) closely related to CRF12_BF and CRF17_BF and more distantly to CRF38_BF and CRF44_BF (Fig. 2). The tree also showed that BOL0137, belonging to the Bolivian cluster, is phylogenetically related to the Euro-Bolivian cluster, a relationship which was not apparent in the tree of the Pr-RT segment. The clustering of the viruses in the BF cluster and their segregation from other South American CRF_BFs was also supported in a 95% confidence network constructed with SplitsTree4 program (Supplementary Fig. S3).

**Figure 3:**
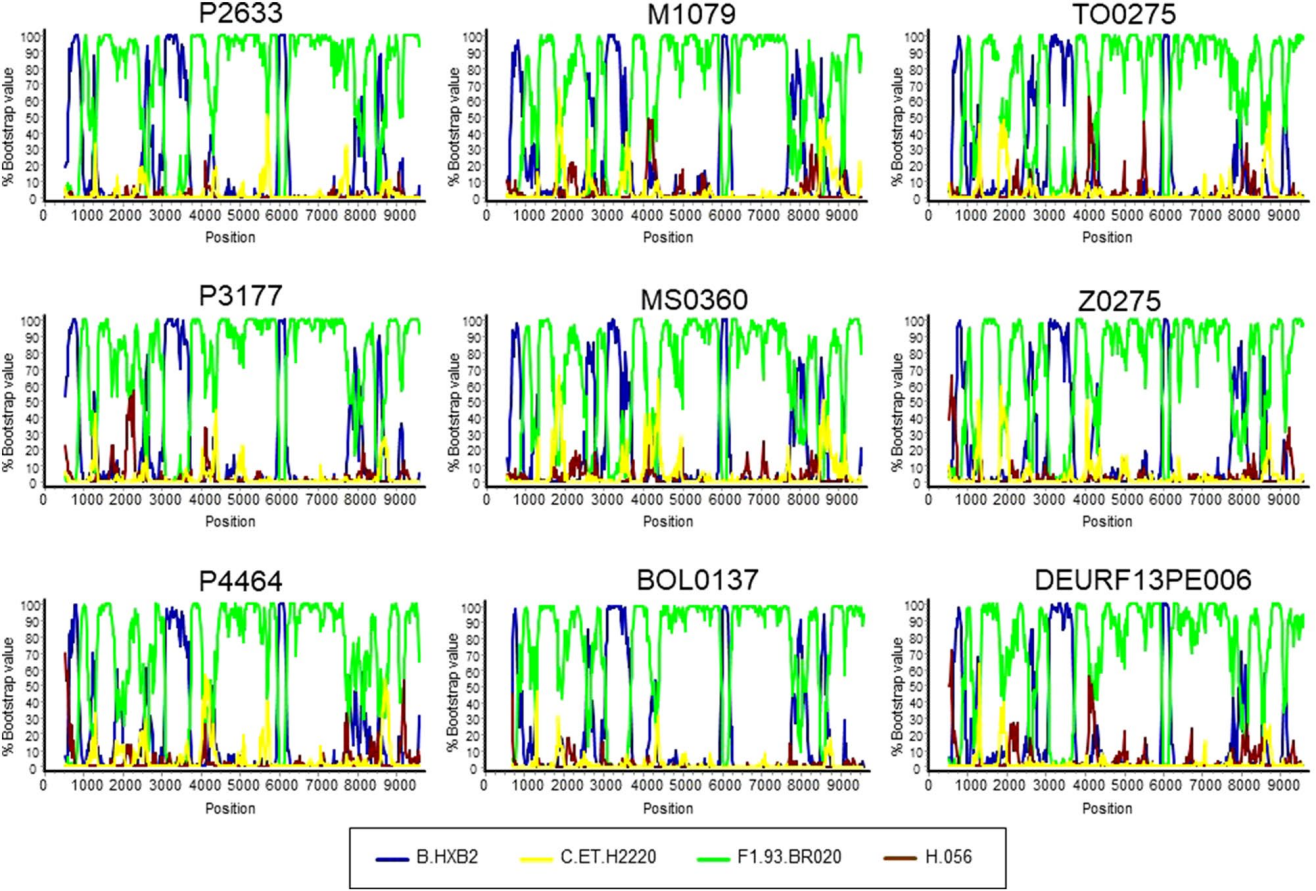
Bootscan analyses of 9 NFLG genomes of the BF cluster. The horizontal axis represents the position in the HXB2 genome of the midpoint of a 250 nt window moving in 20 nt increments and the vertical axis represents bootstrap values supporting clustering with subtype reference sequences.

The mosaic structures of the 9 NFLG sequences were analyzed through bootscan analyses, which showed a complex BF1 recombinant pattern, with highly similar structures and genomes predominantly of F1 subsubtype (Fig. 3). Two ~ 7 kb-long sequences from viruses collected in UK^57^ branching in the BF cluster also showed mosaic structures highly similar to those of the NFLG (Supplementary Fig. S4).

We examined the intersubtype transitions around breakpoints detected by bootscanning by looking at similarities between viruses in the BF cluster and the 75% consensuses of the B and F1 parental clades in the positions where they differ (Supplementary Fig. S5). We found agreement in these transitions among the viruses of the BF cluster, with some minor differences explained by mutations occurring near the breakpoints due to viral evolution since emergence of their common ancestor.

**Figure 4:**
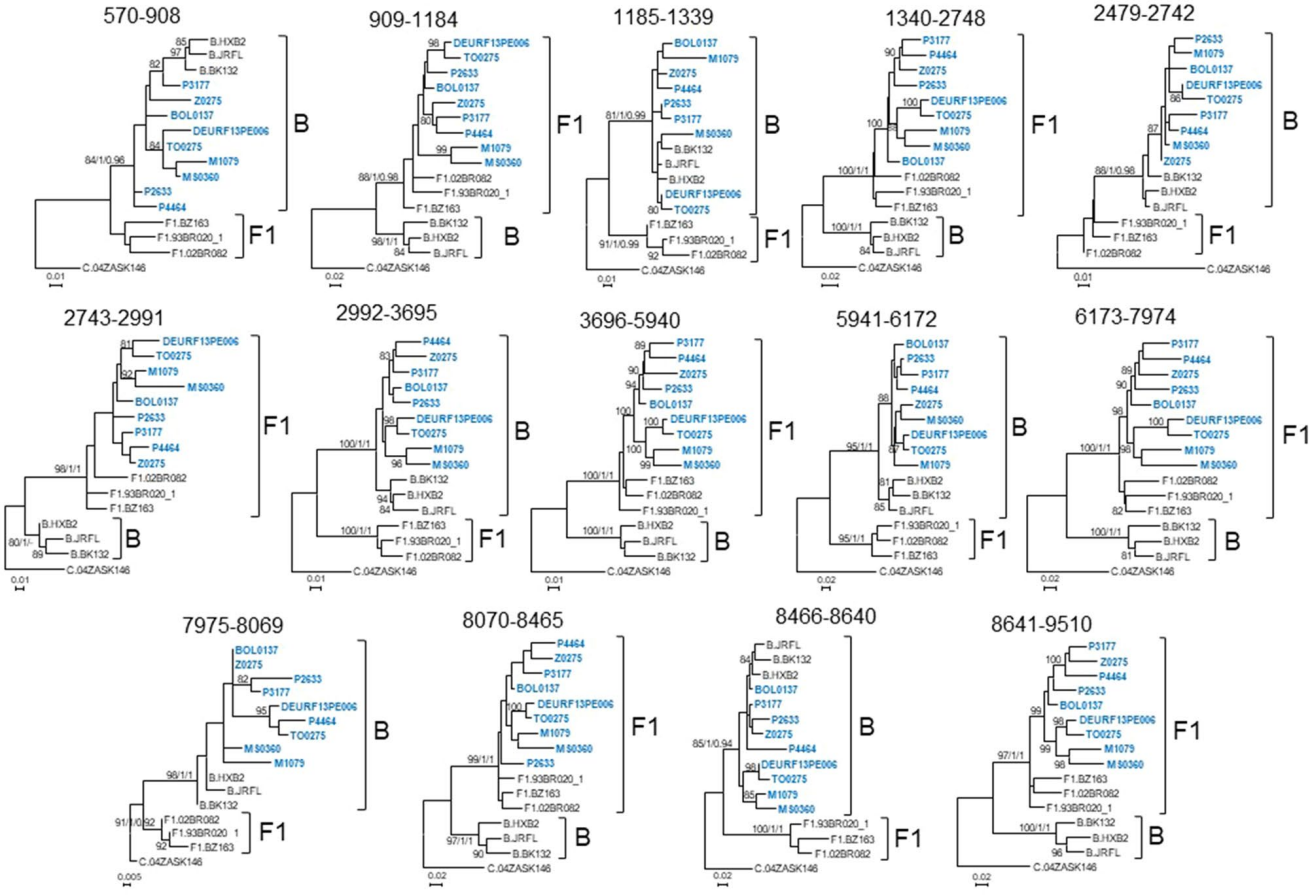
Phylogenetic trees of genome segments of the BF cluster. HXB2 positions delimiting the analyzed segments are indicated on top of the trees. Sequence names of viruses of the BF cluster are in blue. Names of subtype references are preceded by the corresponding subtype. Node supports of B and F1 clades are indicated, in this order, as ultrafast bootstrap value/aLRT SH-like support/posterior probability, which were obtained with IQ-Tree, PhyML, and MrBayes programs, respectively. For the other nodes, only ultrafast bootstrap values are indicated. Only bootstrap values ≥ 80%, aLRT SH-like values ≥ 0.9, and posterior probabilities ≥ 0.9 are shown.

To further examine the subtype assignment of the recombinant segments identified with bootscanning, we analyzed them with ML trees constructed with IQ-Tree and PhyML and with Bayesian trees constructed with MrBayes. Prior to these analyses, the existence of adequate phylogentic signal in three short (< 200 nt) recombinant segments, of subtype B in bootscan analyses (HXB2 positions 1085–1340, 7975–8069, and 8466–8640), was analyzed with likelihood mapping. Since our interest in these analyses was solely to determine the phylogenetic relationship of the viruses in the BF cluster with B and F1 subtype references, likelihood mapping analyses were performed in alignments including subtype references and only one BF virus at a time. The results showed that there was adequate phylogenetic signal in the 3 segments to determine phylogenetic relationship of each virus in the BF cluster with subtype references, with > 70% fully resolved quartets in all cases (Supplementary Table 2). The phylogenetic trees of recombinant segments confirmed the subtype assignments inferred with bootscan analyses, which were coincident among all viruses in the BF cluster (Fig. 4). In the Bayesian tree of the 8466–8640 fragment, the posterior probability support for the assemblage of viruses of the BF cluster with subtype B references was 0.94; however, none of the trees in the posterior (optimal) distribution, clustered the BF viruses with F1 references. The coincidence of subtype assignments among all viruses of the BF cluster included the short segment in the *gp41-tat* overlap (HXB2 positions 8466–8640) in which all viruses, except P4464 and DEURF13PE006, appeared to be of subtype B in bootscan analyses, with phylogenetic trees showing that all of them were of subtype B in this segment (Fig. 4). The phylogenetic trees also showed that the short subtype B segment around nt 8000 (HXB2 positions 7975–8069) was also found in CRF17_BF and CRF38_BF viruses (Supplementary Fig. S6).

Intersubtype breakpoint locations were also analyzed with GARD, RDP4, and jpHMM. The breakpoint locations determined by these programs are shown in Supplementary Table 3. These were generally consistent with the analyses of bootscanning and phylogenetic trees of partial segments, although jpHMM failed to detect breakpoints delimiting two short subtype B segments in *pol* and *env* in all viruses, and some breakpoints in some viruses failed to be detected by some programs. Importantly, no breakpoint failed to be detected by all three programs.

Thus, these analyses show that viruses of the BF cluster have a coincident mosaic structure, which exhibits some similarity to those of CRF12_BF and CRF17_BF, but differs from both in the presence of a short subtype B segment in *gag,* absent from CRF12_BF and CRF17_BF, and from CRF12_BF also in the presence of a short subtype B fragment in *env* around HXB2 position 8000, which is absent from CRF12_BF (Supplementary Fig. S6). Additionally, they also differ in breakpoint positions in p17^gag^, RT and *vpu* (Supplementary Fig. S5), where B-F1 transitions occur between nt positions 889–928, 2678–2807, and 6166–6179 in viruses of the BF cluster vs. 940–961, 2609–2652, and 6193–6235 in CRF12_BF, with transitions in CRF17_BF coinciding with those of CRF12_BF in p17^gag^ and RT and being substantially displaced in the 3’ direction in *vpu*.

**Figure 5:**
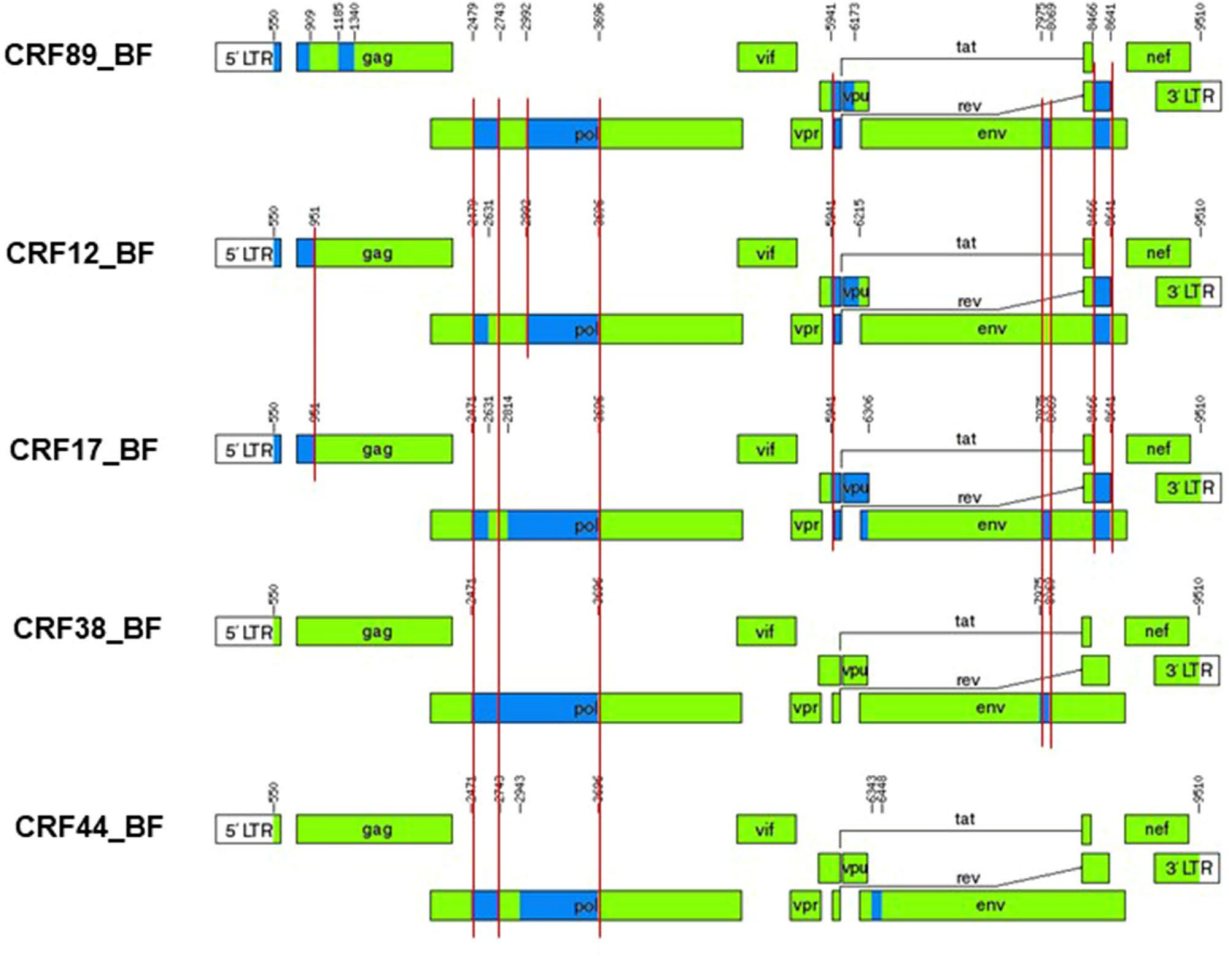
Mosaic structure of CRF89_BF compared to those of CRF12_BF, CRF17_BF, CRF38_BF, and CRF44_BF. Intersubtype breakpoints were determined as the midpoint of B-F1 75% consensus transitions (Supplementary Fig. S5). B and F1 fragments are shown in blue and green, respectively. Vertical red lines indicate coincident breakpoints. Positions correspond to the HXB2 genome.

These results, therefore, show that NFLG sequences of viruses of a BF recombinant cluster group in a clade separate from other CRFs and exhibit a coincident and distinctive mosaic structure, indicating that they represent a new HIV-1 CRF, which was designated CRF89_BF. The mosaic structure of CRF89_BF inferred from bootscan analyses, ML phylogenetic trees of partial segments, analyses with GARD, RDP4, and jpHMM, and intersubtype consensus transitions around breakpoints detected by these methods (Fig. 5) indicates that it is predominantly of subtype F, with 13 breakpoints delimiting 7 subtype B and 7 subtype F segments. Its close relationship with CRF12_BF and CRF17_BF and more distant relationship with CRF38_BF and CRF44_BF are supported by phylogenetic clustering (Fig. 2) and coincidences in 6, 7, 4, and 3 breakpoints, respectively (Fig. 5).

We analyzed amino acid residues found in all or most of the CRF89_BF viruses and absent or uncommon in the related CRF_BFs, numbers 12, 17, 38, and 44, and in the parental Brazilian F1 strain. Twelve characteristic CRF89_BF residues were found, distributed in Gag, Pol, Tat, Rev, and Vif proteins (Supplementary Table 4).

**Figure 6:**
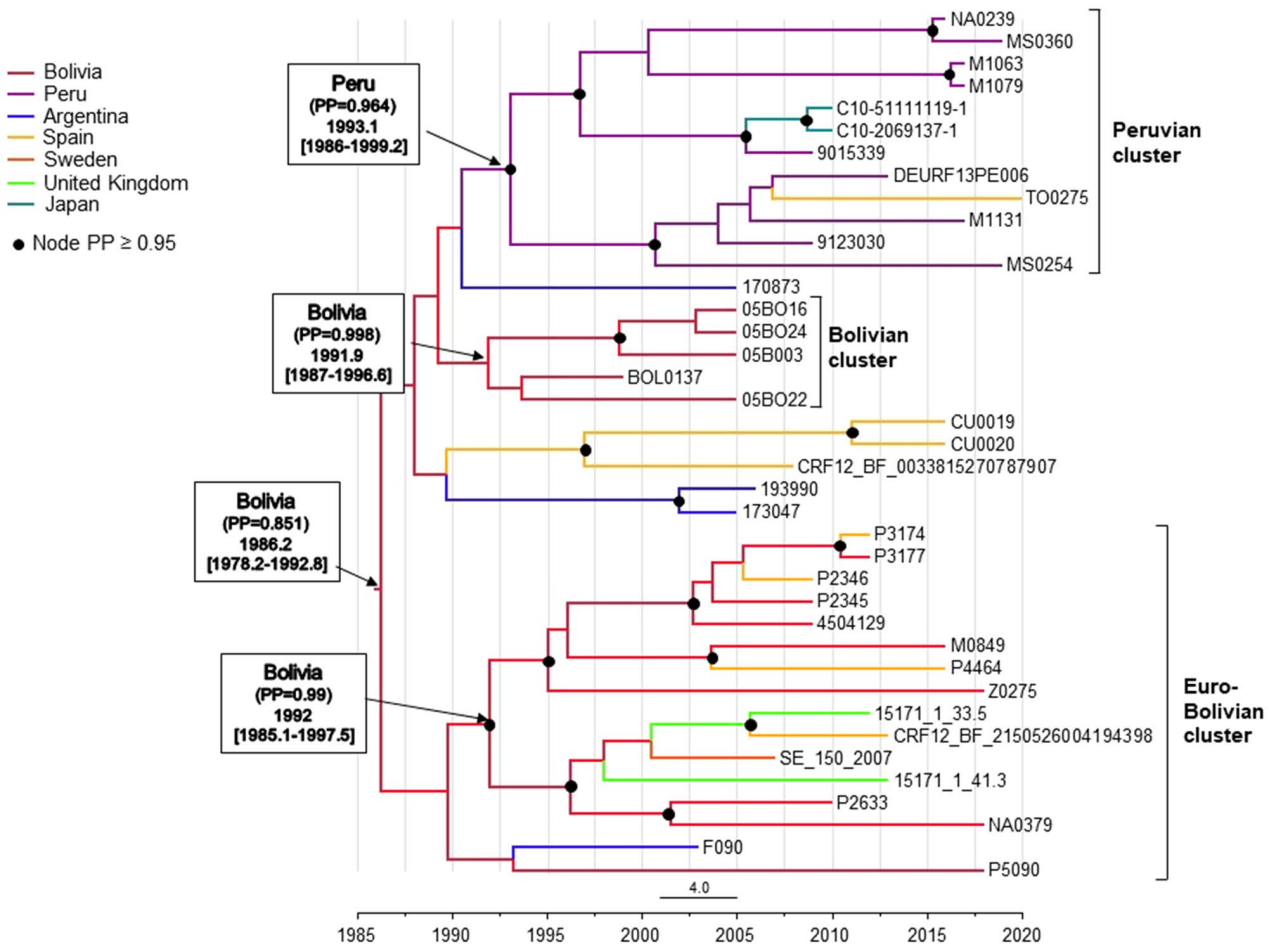
Maximum clade credibility tree of CRF89_BF Pr-RT sequences. Branch colors indicate, for terminal branches, country of sample collection or, if known, of origin of the individual, and for internal branches, the most probable location country of the subtending node, according to the legend on the left. Nodes supported by PP ≥ 0.95 are indicated with filled circles. Most probable countries at the root of the tree and of the three major clusters are indicated, together with PP supporting the locations and tMRCA (mean values, with 95% HPD intervals in parentheses).

We estimated the time of emergence and country of origin of the MRCA of CRF89_BF using Pr-RT sequences and a Bayesian coalescent method. Since the CRF89_BF alignment lacked a sufficient temporal signal, we used as a prior parameter a substitution rate estimated from 65 CRF12_BF sequences, which exhibited an adequate temporal signal (Supplementary Fig. S1). For the BEAST analysis, the country of origin of the individual, when known, was used. This was done because we found no definitive evidence of the epidemic spread of CRF89_BF in Spain (as reflected in clustering among Spanish individuals, which was seen only in two individuals—CU0019 and CU0020—residing in the same city), and, therefore, we assumed that Bolivian and Peruvian immigrants (who represented all foreign-born individuals in the data set) had probably acquired HIV-1 in their countries of origin. For individuals whose country of origin was unknown, country of sample collection was used as location trait. According to this analysis (Fig. 6), the mean estimated time of the MRCA (tMRCA) of CRF89_BF was 1986 (95% HPD, 1978–1992) and its most probable location was Bolivia (PP = 0.851), with the second most probable location being Argentina (PP = 0.097). Estimated times and locations of MRCAs of clusters were 1992 (1985–1997) and Bolivia (PP = 0.99) for the Euro-Bolivian cluster; 1991 (1987–1996) and Bolivia (PP = 0.998) for the Bolivian cluster; and 1993 (1986–1999) and Peru (PP = 0.964) for the Peruvian cluster. Since we could not rule out the possibility that some subclusters of recent origin comprising samples collected in Spain reflected transmissions within the country, we performed an additional BEAST analysis in which the most recently diagnosed infections of two subclusters, comprising samples M1063, M1079 and NA0239, MS0360, respectively, of the Peruvian cluster and 4 infections from the city of Bilbao, P2345, P2346, P3174, and P3177, grouping in a sublcuster in the Euro-Bolivian cluster were assumed to have been acquired in Spain. This analysis also supported an origin of CRF89_BF in Bolivia, although with a lower PP (0.75), and an origin of Euro-Bolivian and Peruvian clusters in Bolivia (PP = 0.87) and Peru (PP = 0.96), respectively (Supplementary Fig. S4).

Among Pr-RT sequences deposited at the HIV Sequence Database from samples collected in Bolivia in 2005 (all from La Paz)^25^, 4 (13.3%) of 30 were of CRF89_BF, which represented 4 (44.4%) of 9 BF recombinant viruses collected that year. By contrast, none of the 21 samples collected in Bolivia in 1996 (from La Paz, Cochabamba, and Santa Cruz), was of CRF89_BF. Among HIV-1-infected Bolivian individuals residing in Spain studied by us sequenced in Pr-RT, 7 (17.9%) of 39 were infected with CRF89_BF viruses, which represented 70% (7 of 10) infections with BF recombinant viruses.

## Discussion

The HIV-1 epidemic in Argentina and the neighboring countries of Uruguay, Chile, Paraguay, and Bolivia is characterized by the cocirculation of B subtype and BF1 recombinant viruses^16–26^. Most of the BF1 recombinant forms in these countries appear to derive from a common recombinant ancestor, as inferred from coincident breakpoints and clustering in phylogenetic trees^17,18,23,27–29^. As the subtype F fragments of these recombinants cluster with viruses of the F subtype strain circulating in Brazil and there is no evidence of the circulation of this strain in other South American countries, it has been proposed that the common ancestor of these recombinants might have originated in Brazil. Subsequent recombination events would have given rise to a great diversity of recombinant forms^17,29^, some of which became circulating, of which CRF12_BF, CRF17_BF, CRF38_BF, and CRF44_BF had been identified previously^17,18,23,27,28^. Due to their common ancestry and similarity in recombination structures, all these viruses have been proposed to constitute a CRF “family”^31,32^ (similarly, other CRF families could be the CRF_BGs from Cuba, numbers 20, 23, and 24^59^, CRF_BGs from Spain and Portugal, numbers 14 and 73^60^, and CRF_01Bs from Malaysia, numbers 33, 53, 58, and 74^61,62^). The first to be identified in the CRF_BF family from the Southern Cone of South America was CRF12_BF, which is widely circulating in Argentina and Uruguay^16–18^ and in lower proportions in Chile^26^, Paraguay^24^, and Bolivia^25^. The second was CRF17_BF, representing a small proportion of infections in Argentina, Paraguay, and Bolivia^27^. The two other members of the family, CRF38_BF and CRF44_BF, were identified in Uruguay^23^ and Chile^28^, respectively. In a molecular epidemiological study in Bolivia, with samples collected in 1996 and 2005, a cluster of 4 BF recombinant viruses branching apart and differing in mosaic structure from CRF12_BF was identified among samples collected in the capital city of La Paz in 2005. The authors proposed that it could represent a new CRF of the CRF12_BF family^25^. Here, we show that this cluster [comprising the 4 viruses collected in 2005 and a fifth virus collected in 1999^18^ (Fig. 1)] forms part of a larger cluster, comprising 39 viruses collected in two other South American countries (Peru and Argentina), three European countries (Spain, United Kingdom, and Sweden), and Japan, with samples collected in Spain representing a majority, although most of them are from Bolivian or Peruvian individuals (Fig. 1). We show through the analysis of 9 NFLG sequences, 7 of them newly derived from samples collected in Spain and two from databases from samples collected in Bolivia and Peru, that the identified cluster represents a new CRF derived from subtypes B and F1, designated CRF89_BF (Figs. 2 and 3). This CRF is closely related to CRF12_BF and CRF17_BF, as deduced from multiple breakpoint coincidences and close phylogenetic clustering, and more distantly to CRF38_BF and CRF44_BF. CRF89_BF has a complex mosaic structure with 13 breakpoints, delimiting 7 subtype F and 7 subtype B fragments. One of the subtype B segments, in *gag*, is absent from CRF12_BF and related CRFs and another segment in *env* is absent from CRF12_BF, but found in CRF17_BF and CRF38_BF. Breakpoint coincidence with different CRF_BFs from the Southern Cone suggests a complex scenario of BF recombinant generation in this area through successive rounds of recombination with subtype B viruses, as previously proposed^28^. However, it seems unlikely that CRF89_BF derives from CRF12_BF or CRF17_BF, since in the NFLG phylogenetic tree the CRF89_BF clade is not nested within CRF12_BF or CRF17_BF radiations, but forms a separate clade (Fig. 2), and exhibits several differences in breakpoint locations from both CRFs (Supplementary Fig. S5).

In a phylogenetic tree of Pr-RT, CRF89_BF comprised three major clusters. One comprised exclusively samples collected in Western Europe (Spain, UK, and Sweden); however, out of 10 individuals with data on country of origin (all residing in Spain), 8 were Bolivian and only 2 were Spanish, whose viruses branch interspersed among those from Bolivian individuals. Therefore, it seems reasonable to assume that this cluster (which was designated Euro-Bolivian cluster) originated and spread initially in Bolivia, and its finding in Western Europe reflects the importation of infections acquired in Bolivia rather than local circulation of CRF89_BF. Otherwise, clustering of CRF89_BF strains among native European individuals would be expected but was not seen. Failure to identify viruses collected in Bolivia within the Euro-Bolivian cluster may be due to the low number of HIV-1 sequences from Bolivia available in public databases. A second CRF89_BF cluster comprises all five samples collected in Bolivia, all from La Paz. In a phylogenetic tree of NFLG, one virus of this cluster is closely related to viruses of the Euro-Bolivian cluster. The third cluster comprises 3 sequences from Peru, 6 from Peruvians residing in Spain, 1 from a Spaniard residing in Spain, and two from Japan, the last ones closely related to a Peruvian virus. Similarly to the case of the Euro-Bolivian cluster, we assume that this cluster represents a variant originated and circulating in Peru and that its presence in Spain and Japan probably reflects the importation of infections acquired in Peru, rather than the local circulation of CRF89_BF. It is interesting to point out that although a small proportion of HIV-1 BF recombinant viruses have been identified in Peru (approximately 2%^19,63^), no evidence has been published of their circulation among the local Peruvian population. Therefore, the results presented here would be the first evidence indicating that an HIV-1 BF1 recombinant form, in this case CRF89_BF, is most likely circulating in Peru. It is also interesting to note that although heterosexual transmission is predominant among CRF89_BF infections, all 4 infections with information on transmission route in a subcluster of 5 individuals within the Peruvian cluster were found in MSM. This reflects the circulation of CRF89_BF among Peruvian MSM and the linkage between HIV-1 heterosexual and MSM transmission networks. A similar linkage was observed in a CRF02_AG cluster in Spain, although in this case the spread was from an MSM to a heterosexual network^64^.

According to phylodynamic estimations, CRF89_BF probably emerged in Bolivia around the mid-1980s, with its major clusters emerging around the first half of the 1990s, two of them in Bolivia and one in Peru (Fig. 6). These estimations were done assuming that CRF89_BF infections in Bolivian and Peruvian individuals residing in Spain acquired their infections in their country of origin, which seems a reasonable assumption, as discussed above. However, since we could not rule out that subclusters of more recent origin comprising viruses sampled in Spain reflected local transmissions, a second analysis assuming HIV-1 acquisition in Spain of the most recently diagnosed infections of subclusters comprising Bolivian or Peruvian individuals was performed, yielding similar results (Supplementary Fig. S4). The MRCA of CRF89_BF, according to our estimations, would be around 11 years more recent than that of CRF12_BF (Supplementary Fig. S8). However, we cannot rule out an earlier emergence of CRF89_BF, since estimations could have changed with a more representative sampling of Bolivian HIV-1.

In Bolivia, CRF89_BF was detected in only 5 samples from La Paz, 4 collected in 2005 and 1 in 1997. In 2005, CRF89_BF represented 13.3% HIV-1 samples collected in La Paz sequenced for Pr-RT. However, given the low proportion of Bolivian HIV-1 strains sequenced and the fact that no sequences from samples collected after 2005 are available in public databases, the current prevalence of CRF89_BF in Bolivia and its geographical spread in that country cannot be accurately estimated. Considering that in one of the major CRF89_BF clusters 8 of 10 viruses, all of which were collected in Europe, were from Bolivian individuals, and that 18% of the HIV-1-infected Bolivian individuals residing in Spain studied by us harbored CRF89_BF viruses, we hypothesize that CRF89_BF could be circulating widely in some areas of Bolivia.

The identification of CRF89_BF infections in Spain and other European countries, mainly in South American immigrants, reflects the increasing relation between the South American and European HIV-1 epidemics, which is also reflected in the expansion in Western Europe of clusters of South American strains of subtypes C^64–68^ and F1^33,69–71^, of CRF12_BF^72^, and of CRF17_BF^67^, and in the identification in Western Europe of CRFs derived from parental strains of South American ancestry^73–75^.

The identification of CRF89_BF and other CRFs in NFLG sequences is relevant for molecular epidemiological studies because it allows for the proper characterization of HIV-1 strains circulating in different geographic areas and population groups. In this regard, some CRF89_BF viruses were misclassified as CRF12_BF viruses in GenBank submissions (accessions MF403410, MF403416). Such misclassification may not be irrelevant, since, even though both CRFs exhibit similar mosaic structures, they are not identical and form separate clades. It should also be pointed out that even relatively minor genetic differences in viral genomes may result in important biological differences. Examples in HIV-1 are CXCR4 coreceptor usage in CRF14_BG, which is associated with only four amino acid residues in the Env V3 loop^76^, all or most of which are absent in viruses of the closely related CRF73_BG^60^, which has a very similar, but not identical, mosaic structure; and differences in pathogenic potential or therapeutic response associated with clusters within HIV-1 genetic forms^77,78^. The identification of CRF89_BF may be also relevant for the development and testing of vaccines intended for use in areas where this CRF circulates, considering the correlation of susceptibility to protective immune responses with HIV-1 clades and with intraclade genetic diversity^5^.

## Supplementary Information

Supplementary Information.

## Data Availability

Sequences are deposited in GenBank under accessions KX818199, KX818200, MW344906-MW344922, and MW802822-MW802825.

## References

[1] Shriner D, Rodrigo AG, Nickle DC, Mullins JI (2004). Pervasive genomic recombination of HIV-1 in vivo. Genetics.

[2] Charpentier C, Nora T, Tenaillon O, Clavel F, Hance AJ (2006). Extensive recombination among human immunodeficiency virus type 1 quasispecies makes an important contribution to viral diversity in individual patients. J. Virol..

[3] Perelson AS, Neumann AU, Markowitz M, Leonard JM, Ho DD (1996). HIV-1 dynamics in vivo: Virion clearance rate, infected cell life-span and viral generation time. Science.

[4] Jung A (2002). Recombination: Multiply infected spleen cells in HIV patients. Nature.

[5] Hraber, P. *et al.* Impact of clade, geography, and age of the epidemic on HIV-1 neutralization by antibodies. *J. Virol.* **88**, 12623–12643. 10.1128/JVI.01705-14 (2014). 10.1128/JVI.01705-14 PMC4248897 25142591

[6] Gundlach BR (2000). Evidence for recombination of live, attenuated immunodeficiency virus vaccine with challenge virus to a more virulent strain. J. Virol..

[7] Moradigaravand, D. *et al*. Recombination accelerates adaptation on a large-scale empirical fitness landscape in HIV-1. *PLoS Genet.* **10**, e1004439. 10.1371/journal.pgen.1004439 (2014). 10.1371/journal.pgen.1004439 PMC4072600 24967626

[8] Ritchie, A.J. *et al*. Recombination-mediated escape from primary CD8+ T cells in acute HIV-1 infection. Retrovirology **11**, 69; 10.1186/s12977-014-0069-9 (2014). 10.1186/s12977-014-0069-9 PMC4180588 25212771

[9] Arenas M, Lorenzo-Redondo R, López-Galíndez C (2016). Influence of mutation and recombination on HIV-1 *in vitro* fitness. Mol. Phylogenet. Evol..

[10] Streeck H (2008). Immune-driven recombination and loss of control after HIV superinfection. J. Exp. Med..

[11] Song, H. *et al*. Tracking HIV-1 recombination to resolve its contribution to HIV-1 evolution in natural infection. *Nat. Commun.* **9**, 1928. 10.1038/s41467-018-04217-5 (2018). 10.1038/s41467-018-04217-5 PMC5954121 29765018

[12] Nora T (2007). Contribution of recombination to the evolution of human immunodeficiency viruses expressing resistance to antiretroviral treatment. J. Virol..

[13] Nájera R, Delgado E, Pérez-Álvarez L, Thomson MM (2002). Genetic recombination and its role in the development of the HIV-1 pandemic. AIDS.

[14] Hemelaar J (2020). Global and regional epidemiology of HIV-1 recombinants in 1990–2015: A systematic review and global survey. Lancet HIV.

[15] Robertson DL (2000). HIV-1 nomenclature proposal. Science.

[16] Thomson MM (2000). Widespread circulation of a B/F intersubtype recombinant form among HIV-1-infected individuals in Buenos Aires, Argentina. AIDS.

[17] Thomson MM (2002). Diversity of mosaic structures and common ancestry of human immunodeficiency virus type 1 BF intersubtype recombinant viruses from Argentina revealed by analysis of near full-length genome sequences. J. Gen. Virol..

[18] Carr JK (2001). Diverse BF recombinants have spread widely since the introduction of HIV-1 into South America. AIDS.

[19] Hierholzer J (2002). Molecular epidemiology of HIV type 1 in Ecuador, Peru, Bolivia, Uruguay and Argentina. AIDS Res. Hum. Retroviruses.

[20] Quarleri JF (2004). HIV type 1 BF recombinant strains exhibit different pol gene mosaic patterns: Descriptive analysis from 284 patients under treatment failure. AIDS Res. Hum. Retroviruses.

[21] Dilernia DA (2007). HIV type 1 genetic diversity surveillance among newly diagnosed individuals from 2003 to 2005 in Buenos Aires, Argentina. AIDS Res. Hum. Retroviruses.

[22] Pando MA (2008). Human immunodeficiency virus and tuberculosis in Argentina: Prevalence, genotypes and risk factors. J. Med. Microbiol..

[23] Ruchansky D, Casado C, Russi JC, Arbiza JR, López-Galíndez C (2009). Identification of a new HIV type 1 circulating recombinant form (CRF38_BF1) in Uruguay. AIDS Res. Hum. Retroviruses.

[24] Aguayo N (2008). Epidemiological and molecular characteristics of HIV-1 infection among female commercial sex workers, men who have sex with men and people living with AIDS in Paraguay. Rev. Soc. Bras. Med. Trop..

[25] Guimaraes, M. L., Velarde-Dunois, K. G., Segurondo, D. & Morgado, M. G. The HIV-1 epidemic in Bolivia is dominated by subtype B and CRF12_BF "family" strains. *Virol. J.* **9**, 19. 10.1186/1743-422X-9-19 (2012). 10.1186/1743-422X-9-19 PMC3285048 22248191

[26] Ríos M (2007). Antiretroviral drug resistance and phylogenetic diversity of HIV-1 in Chile. J. Med. Virol..

[27] Aulicino PC (2012). Characterization of full-length HIV-1 CRF17_BF genomes and comparison to the prototype CRF12_BF strains. Infect. Genet. Evol..

[28] Delgado E (2010). Identification of a new HIV type 1 BF intersubtype circulating recombinant form (CRF44_BF) in Chile. AIDS Res. Hum. Retroviruses.

[29] Sierra M (2005). The analysis of near full-length genome sequences of human immunodeficiency virus type 1 BF intersubtype recombinant viruses from Chile, Venezuela and Spain reveals their relationship to diverse lineages of recombinant viruses related to CRF12_BF. Infect. Genet. Evol..

[30] Cevallos, C. G. *et al*. Genomic characterization and molecular evolution analysis of subtype B and BF recombinant HIV-1 strains among Argentinean men who have sex with men reveal a complex scenario. *PLoS One* **12**, e0189705. 10.1371/journal.pone.0189705 (2017). 10.1371/journal.pone.0189705 PMC5731684 29244833

[31] Thomson MM, Nájera R (2005). Molecular epidemiology of HIV-1 variants in the global AIDS pandemic: an update. AIDS Rev..

[32] Zhang, M. et al. The role of recombination in the emergence of a complex and dynamic HIV epidemic. *Retrovirology* **7**, 25, 10.1186/1742-4690-7-25 (2010). 10.1186/1742-4690-7-25 PMC2855530 20331894

[33] Delgado, E. *et al*. Phylogeny and phylogeography of a recent HIV-1 subtype F outbreak among men who have sex with men in Spain deriving from a cluster with a wide geographic circulation in Western Europe. *PLoS One* **10**, e0143325, 10.1371/journal.pone.0143325 (2015). 10.1371/journal.pone.0143325 PMC4658047 26599410

[34] Delgado E (2002). Identification of a newly characterized HIV-1 BG intersubtype circulating recombinant form in Galicia, Spain, which exhibits a pseudotype-like virion structure. J. Acquir. Immune Defic. Syndr..

[35] Katoh K, Standley DM (2013). MAFFT multiple sequence alignment software version 7: Improvements in performance and usability. Mol. Biol. Evol..

[36] Price, M. N., Dehal, P. S. & Arkin, A. P. FastTree 2—Approximately maximum-likelihood trees for large alignments. *PLoS One* **5**, e9490, 10.1371/journal.pone.0009490 (2010). 10.1371/journal.pone.0009490 PMC2835736 20224823

[37] Guindon, S. *et al*. New algorithms and methods to estimate maximum-likelihood phylogenies: Assessing the performance of PhyML 3.0. *Syst. Biol.* **59**, 307–321 (2010). 10.1093/sysbio/syq010 20525638

[38] Nguyen LT, Schmidt HA, von Haeseler A, Minh BQ (2015). IQ-TREE: A fast and effective stochastic algorithm for estimating maximum-likelihood phylogenies. Mol. Biol. Evol..

[39] Kalyaanamoorthy S (2017). ModelFinder: Fast model selection for accurate phylogenetic estimates. Nat. Methods.

[40] Hoang DT (2018). UFBoot2: Improving the ultrafast bootstrap approximation. Mol. Biol. Evol..

[41] Kumar, S., Stecher, G. & Tamura, K. MEGA7: Molecular evolutionary genetics analysis version 7.0 for bigger datasets. *Mol. Biol. Evol.* **33**, 1870–1874 (2016). 10.1093/molbev/msw054 PMC8210823 27004904

[42] Huson DH, Bryant D (2006). Application of phylogenetic networks in evolutionary studies. Mol. Biol. Evol..

[43] Salminen MO, Carr JK, Burke DS, McCutchan FE (1995). Identification of breakpoints in intergenotypic recombinants of HIV type 1 by bootscanning. AIDS Res. Hum. Retroviruses.

[44] Lole KS (1999). Full-length human immunodeficiency virus type 1 genomes from subtype C-infected seroconverters in India, with evidence of intersubtype recombination. J. Virol..

[45] Guindon S, Gascuel O (2003). PhyML: a simple, fast, and accurate algorithm to estimate large phylogenies by maximum likelihood. Syst. Biol..

[46] Ronquist, F. *et al*. MrBayes 3.2: efficient Bayesian phylogenetic inference and model choice across a large model space. *Syst. Biol.* **61**, 539–542 (2012). 10.1093/sysbio/sys029 PMC3329765 22357727

[47] Strimmer, K. and von Haeseler A. Likelihood-mapping: a simple method to visualize phylogenetic content of a sequence alignment. *Proc. Natl. Acad. Sci. U S A*. **94**, 6815–6819 (1997). 10.1073/pnas.94.13.6815 PMC21241 9192648

[48] Kosakovsky Pond, S. L. *et al*. GARD: a genetic algorithm for recombination detection. *Bioinformatics* **22**, 3096–3098 (2006). 10.1093/bioinformatics/btl474 17110367

[49] Martin, D. P. *et al*. RDP4: Detection and analysis of recombination patterns in virus genomes. *Virus Evol.* **1**, vev003. 10.1093/ve/vev003 (2015). 10.1093/ve/vev003 PMC5014473 27774277

[50] Schultz AK (2009). jpHMM: Improving the reliability of recombination prediction in HIV-1. Nucleic Acids Res..

[51] Drummond, A. J., Suchard, M. A., Xie, D. & Rambaut, A. Bayesian phylogenetics with BEAUti and the BEAST 1.7. *Mol. Biol. Evol.* **29**, 1969–1973 (2012). 10.1093/molbev/mss075 PMC3408070 22367748

[52] Tang MW, Liu TF, Shafer RW (2012). The HIVdb system for HIV-1 genotypic resistance interpretation. Intervirology.

[53] Rambaut, A., Lam, T. T., Max, C. L. & Pybus, O. G. Exploring the temporal structure of heterochronous sequences using TempEst (formerly Path-O-Gen). *Virus Evol.* **2**, vew007. 10.1093/ve/vew007 (2016). 10.1093/ve/vew007 PMC4989882 27774300

[54] Shapiro B, Rambaut A, Drummond AJ (2006). Choosing appropriate substitution models for the phylogenetic analysis of protein-coding sequences. Mol. Biol. Evol..

[55] Drummond AJ, Rambaut A, Shapiro B, Pybus OG (2005). Bayesian coalescent inference of past population dynamics from molecular sequences. Mol. Biol. Evol..

[56] Los Alamos National Laboratory. *HIV Sequence Database*. https://www.hiv.lanl.gov/content/sequence/HIV/mainpage.html.

[57] Yebra G (2018). A high HIV-1 strain variability in London, UK, revealed by full-genome analysis: Results from the ICONIC project. PLoS ONE.

[58] National Center for Biotechnology Information. *GenBank*. https://www.ncbi.nlm.nih.gov/genbank/.

[59] Sierra M (2007). Identification of 3 phylogenetically related HIV-1 BG intersubtype circulating recombinant forms in Cuba. J. Acquir. Immune Defic. Syndr..

[60] Fernández-García, A. *et al*. Identification of an HIV-1 BG intersubtype recombinant form (CRF73_BG), partially related to CRF14_BG, which is circulating in Portugal and Spain. *PLoS One* **11**, e0148549. 10.1371/journal.pone.0148549 (2016). 10.1371/journal.pone.0148549 PMC4765764 26900693

[61] Chow, W. Z. *et al*. A newly emerging HIV-1 recombinant lineage (CRF58_01B) disseminating among people who inject drugs in Malaysia. *PLoS One* **9**, e85250. 10.1371/journal.pone.0085250 (2014). 10.1371/journal.pone.0085250 PMC3898983 24465513

[62] Cheong, H. T. *et al*. Genetic characterization of a novel HIV-1 circulating recombinant form (CRF74_01B) identified among intravenous drug users in Malaysia: Recombination history and phylogenetic linkage with previously defined recombinant lineages. *PLoS One* **10**, e0133883. 10.1371/journal.pone.0133883 (2015). 10.1371/journal.pone.0133883 PMC4510129 26196131

[63] Carrión AG (2009). Molecular characterization of the human immunodeficiency virus type 1 among children in Lima, Peru. AIDS Res. Hum. Retroviruses.

[64] Delgado E (2019). Diverse large HIV-1 non-subtype B clusters are spreading among men who have sex with men in Spain. Front. Microbiol..

[65] de Oliveira, T., Pillay, D. & Gifford, R. J. The HIV-1 subtype C epidemic in South America is linked to the United Kingdom. *PLoS One* **5**, e9311. 10.1371/journal.pone.0009311 (2010). 10.1371/journal.pone.0009311 PMC2824804 20174561

[66] Collaço Verás, N. M ., Gray, R. R., de Macedo Brígido, L. F., Rodrigues, R. & Salemi, M. High-resolution phylogenetics and phylogeography of human immunodeficiency virus type 1 subtype C epidemic in South America. *J. Gen. Virol.* **92**, 1698–1709 (2011). 10.1099/vir.0.028951-0 21450946

[67] Lai A (2014). Phylogenetic analysis provides evidence of interactions between Italian heterosexual and South American homosexual males as the main source of national HIV-1 subtype C epidemics. J. Med. Virol..

[68] Fabeni, L. *et al*. Recent transmission clustering of HIV-1 C and CRF17_BF strains characterized by NNRTI-related mutations among newly diagnosed men in Central Italy. *PLoS One* **10**, e0135325. 10.1371/journal.pone.0135325 (2015). 10.1371/journal.pone.0135325 PMC4535860 26270824

[69] Vinken, L. *et al*. Earlier initiation of antiretroviral treatment coincides with an initial control of the HIV-1 sub-subtype F1 outbreak among men-having-sex-with-men in Flanders, Belgium. *Front. Microbiol.* **10**, 613. 10.3389/fmicb.2019.00613 (2019). 10.3389/fmicb.2019.00613 PMC6443750 30972053

[70] Thomson MM (2012). Rapid expansion of a HIV-1 subtype F cluster of recent origin among men who have sex with men in Galicia, Spain. J. Acquir. Immune Defic. Syndr..

[71] Carvalho A (2015). Analysis of a local HIV-1 epidemic in Portugal highlights established transmission of non-B and non-G subtypes. J. Clin. Microbiol..

[72] Fabeni, L. *et al*. Evaluation of HIV transmission clusters among natives and foreigners living in Italy. *Viruses* **12**, 791. 10.3390/v12080791 (2020). 10.3390/v12080791 PMC7472346 32718024

[73] Fernández-García A (2010). Identification of a new HIV type 1 circulating BF intersubtype recombinant form (CRF47_BF) in Spain. AIDS Res. Hum. Retroviruses.

[74] Struck D (2015). Near full-length characterization and population dynamics of the human immunodeficiency virus type 1 circulating recombinant form 42 (CRF42_BF) in Luxembourg. AIDS Res. Hum. Retroviruses.

[75] Simonetti FR (2014). Identification of a new HIV-1 BC circulating recombinant form (CRF60_BC) in Italian young men having sex with men. Infect. Genet. Evol..

[76] Pérez-Álvarez L (2014). Predominance of CXCR4 tropism in HIV-1 CRF14_BG strains from newly diagnosed infections. J. Antimicrob. Chemother..

[77] Cid-Silva P (2018). Initial treatment response among HIV subtype F infected patients who started antiretroviral therapy based on integrase inhibitors. AIDS.

[78] Song H (2019). Disparate impact on CD4 T cell count by two distinct HIV-1 phylogenetic clusters from the same clade. Proc. Natl. Acad. Sci. U.S.A..

