# Author Correction: Identification of CRF89_BF, a new member of an HIV-1 circulating BF intersubtype recombinant form family widely spread in South America

**DOI:** 10.1038/s41598-021-95679-z

**Published:** 2021-08-03

**Authors:** Elena Delgado, Aurora Fernández-García, Marcos Pérez-Losada, María Moreno-Lorenzo, Ismael Fernández-Miranda, Sonia Benito, Vanessa Montero, Horacio Gil, Silvia Hernáez, Josefa Muñoz, Miren Z. Zubero-Sulibarria, Elena García-Bodas, Mónica Sánchez, Jorge del Romero, Carmen Rodríguez, Luis Elorduy, Elena Bereciartua, Esther Culebras, Icíar Rodríguez-Avial, María Luisa Giménez-Alarcón, Carmen Martín-Salas, Carmen Gómez-González, José J. García-Irure, Gema Cenzual, Ana Martínez-Sapiña, María Maiques-Camarero, Lucía Pérez-Álvarez, Michael M. Thomson

**Affiliations:** 1 HIV Biology and Variability Unit, Centro Nacional de Microbiología, Instituto de Salud Carlos III, Majadahonda, Madrid, Spain; 2 Consortium for Biomedical Research in Epidemiology and Public Health (CIBERESP), Instituto de Salud Carlos III, Madrid, Spain; 3 Department of Biostatistics and Bioinformatics, Computational Biology Institute, Milken Institute School of Public Health, George Washington University, Washington, DC USA; 4 CIBIO-InBIO, Centro de Investigação em Biodiversidade e Recursos Genéticos, Universidade do Porto, Campus Agrário de Vairão, Vairão, Portugal; 5 Hospital Universitario Basurto, Bilbao, Spain; 6 Centro Sanitario Sandoval, Madrid, Spain; 7 Hospital Universitario Cruces, Instituto de Investigación Biocruces, Baracaldo, Vizcaya Spain; 8 Hospital Clínico Universitario San Carlos, Madrid, Spain; 9 Hospital Virgen de La Luz, Cuenca, Spain; 10 Complejo Hospitalario de Navarra, Pamplona, Spain; 11 Hospital Universitario Araba, Vitoria-Gasteiz, Spain; 12 Hospital Reina Sofía, Tudela, Navarra Spain; 13 Hospital Universitario Severo Ochoa, Leganés, Madrid, Spain; 14 Hospital Universitario Miguel Servet, Zaragoza, Spain; 15 Hospital Nuestra Señora del Prado, Talavera de la Reina, Toledo Spain; 16 Present Address: Instituto de Investigación Sanitaria Puerta de Hierro-Segovia de Arana, Majadahonda, Madrid, Spain

Correction to: *Scientific Reports* 10.1038/s41598-021-90023-x, published online 01 June 2021

The original version of this Article omitted the Funding section. The Funding section now reads:

“This work was funded through Acción Estratégica en Salud Intramural (AESI), Instituto de Salud Carlos III, projects “Estudios sobre vigilancia epidemiológica molecular del VIH-1 en España”, PI16CIII/00033, and “Epidemiología molecular del VIH en España y su utilidad para investigaciones biológicas y en vacunas”, PI19CIII/00042; Red de Investigación en SIDA (RIS), Instituto de Salud Carlos III, Subdirección General de Evaluación y Fondo Europeo de Desarrollo Regional (FEDER), Plan Nacional I+D+I, project RD16ISCIII/0002/0004; and scientific agreement with Osakidetza-Servicio Vasco de Salud, Government of Basque Country, MVI 1001/16.”

The original Article has been corrected.

