# Supplementary material for: Identification of CRF89_BF, a new member of an HIV-1 circulating BF intersubtype recombinant form family widely spread in South America
Source: Sci Rep. 2021 Jun 1;11:11442. doi: 10.1038/s41598-021-90023-x (PMC8169922; doi:10.1038/s41598-021-90023-x)
Supplement: Supplementary file 1 — Supplementary Information. [file 41598_2021_90023_MOESM1_ESM.pdf]

Identification of CRF89_BF, a new member of an HIV-1 circulating BF intersubtype recombinant form family widely spread in South America.

Elena Delgado, Aurora Fernández-García, Marcos Pérez-Losada, María Moreno-Lorenzo, Ismael Fernández-Miranda, Sonia Benito, Vanessa Montero, Horacio Gil, Silvia Hernáez, Josefa Muñoz, Miren Z. Zubero-Sulibarria, Elena García-Bodas, Mónica Sánchez, Jorge del Romero, Carmen Rodríguez, Luis Elorduy, Elena Bereciartua, Esther Culebras, Icíar Rodríguez-Avial, María Luisa Giménez-Alarcón, Carmen Martín-Salas, Carmen Gómez-González, José J García-Irure, Gema Cenzual, Ana Martínez-Sapiña, María Maiques-Camarero, Lucía Pérez-Álvarez, and Michael M Thomson.

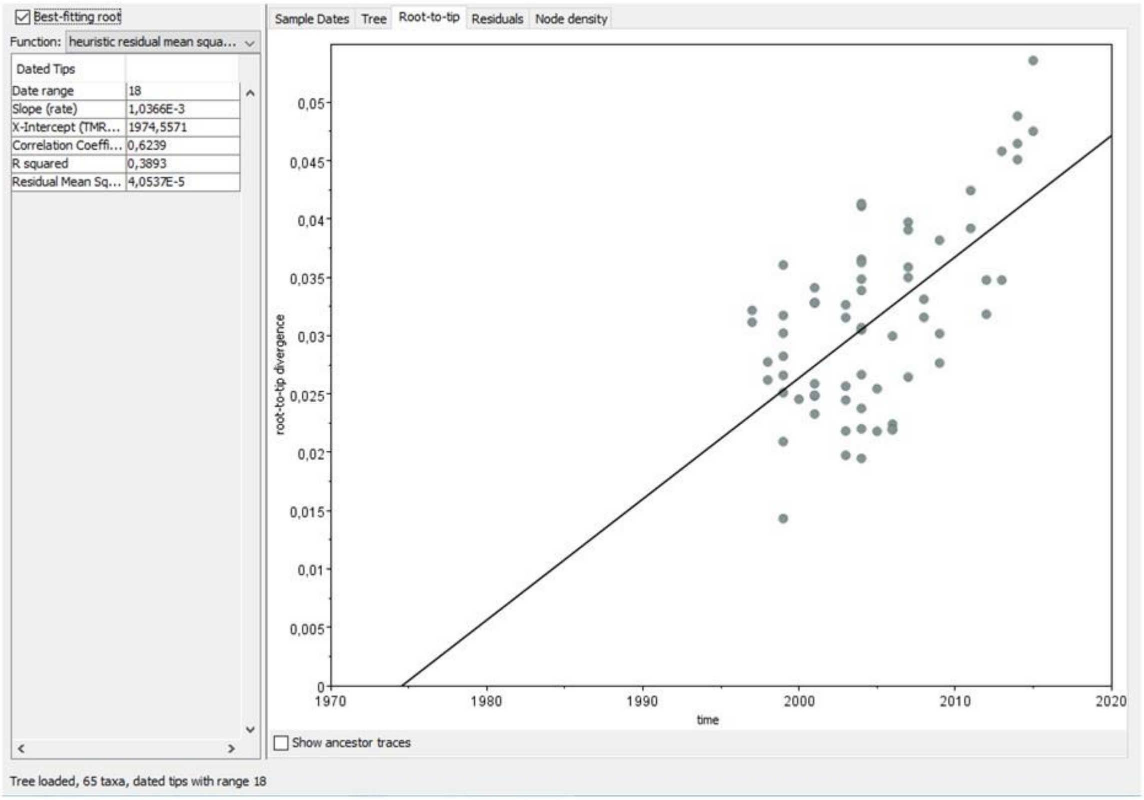

Supplementary Fig. S1. Analysis of root-to-tip divergence vs. year of sample collection of 65 CRF12_BF Pr-RT sequences with TempEst. The analysis was done after removing 8 outlying sequences identified in initial analyses.

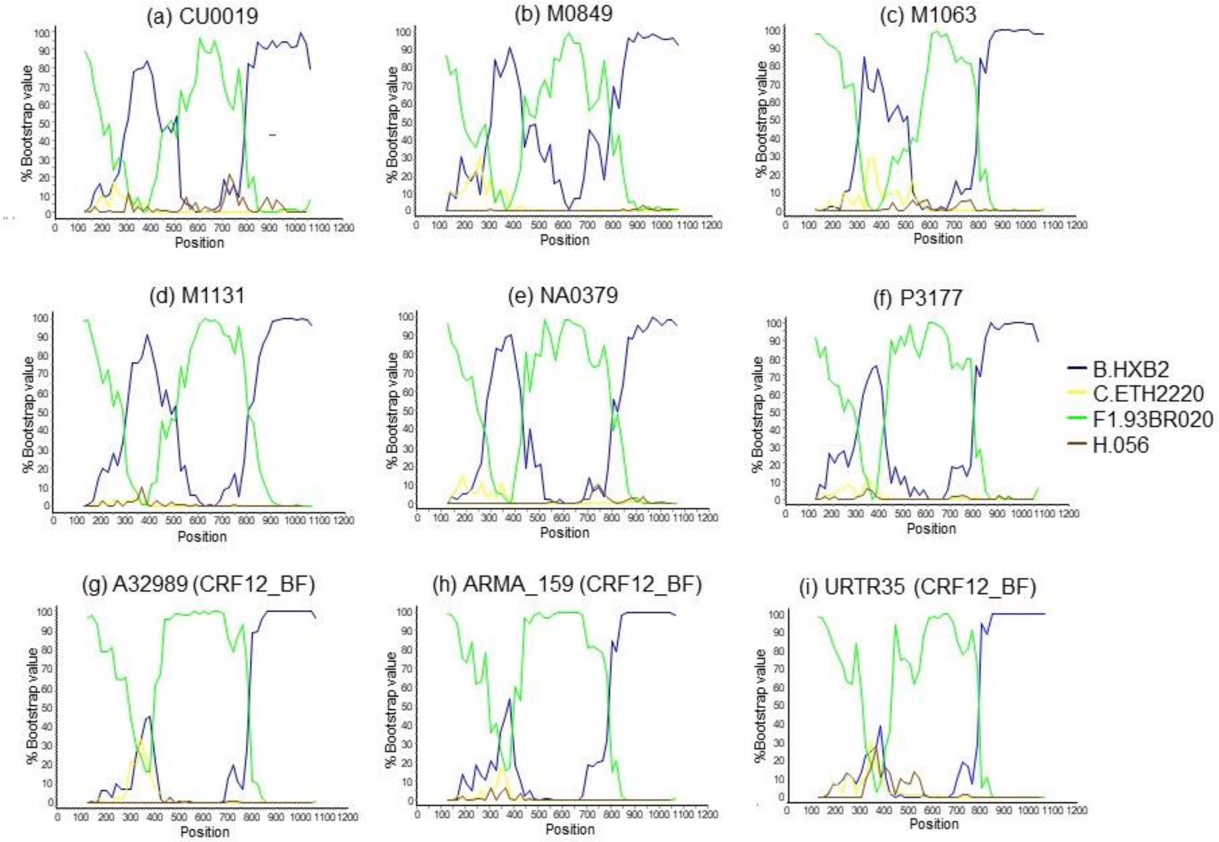

Supplementary Fig. S2. Bootscan analyses of 6 Pr-RT sequences of the BF cluster (a-f) and 3 of CRF12_BF (g-i). The horizontal axis represents the position from the 3' end of protease of the mid-point of a 250 nt window moving in 20 nt increments and the vertical axis represents bootstrap values supporting clustering with subtype reference sequences.

Supplementary Table 1. Clinical data of patients at the time of sample collection.

Sample ID	Plasma viral load (cp/ml)	CD4+ T-cell count (cells/µl)	Clinical stage	Antiretroviral therapy
**CU0019**	1,510	942	A1	No
**CU0020**	66,300	433	A2	No
**M0849**	76,700	450	A2	No
**M1063**	4,069	479	A2	No
**M1079**	8,790	994	A1	No
**M1131**	100,000	285	n.a.	No
**MS0254**	16,330	704	A1	No
**MS0360**	552,000	247	A2	No
**NA0239**	2,360,000	281	C2	No
**NA0379**	3,210,000	39	C1	No
**P2345**	5,440	208	C2	No
**P2346**	356,400	202	A2	No
**P2633**	1,174,897	8	B3	No
**P3174**	10,000,000	1080	B1	No
**P3177**	1,100,000	307	B2	No
**P4464**	270,000	330	A2	No
**P5090**	167,000	445	A2	No
**TO0275**	21,600	469	A2	No
**Z0275**	14,5000	380	A2	No

n.a.: datum not available.

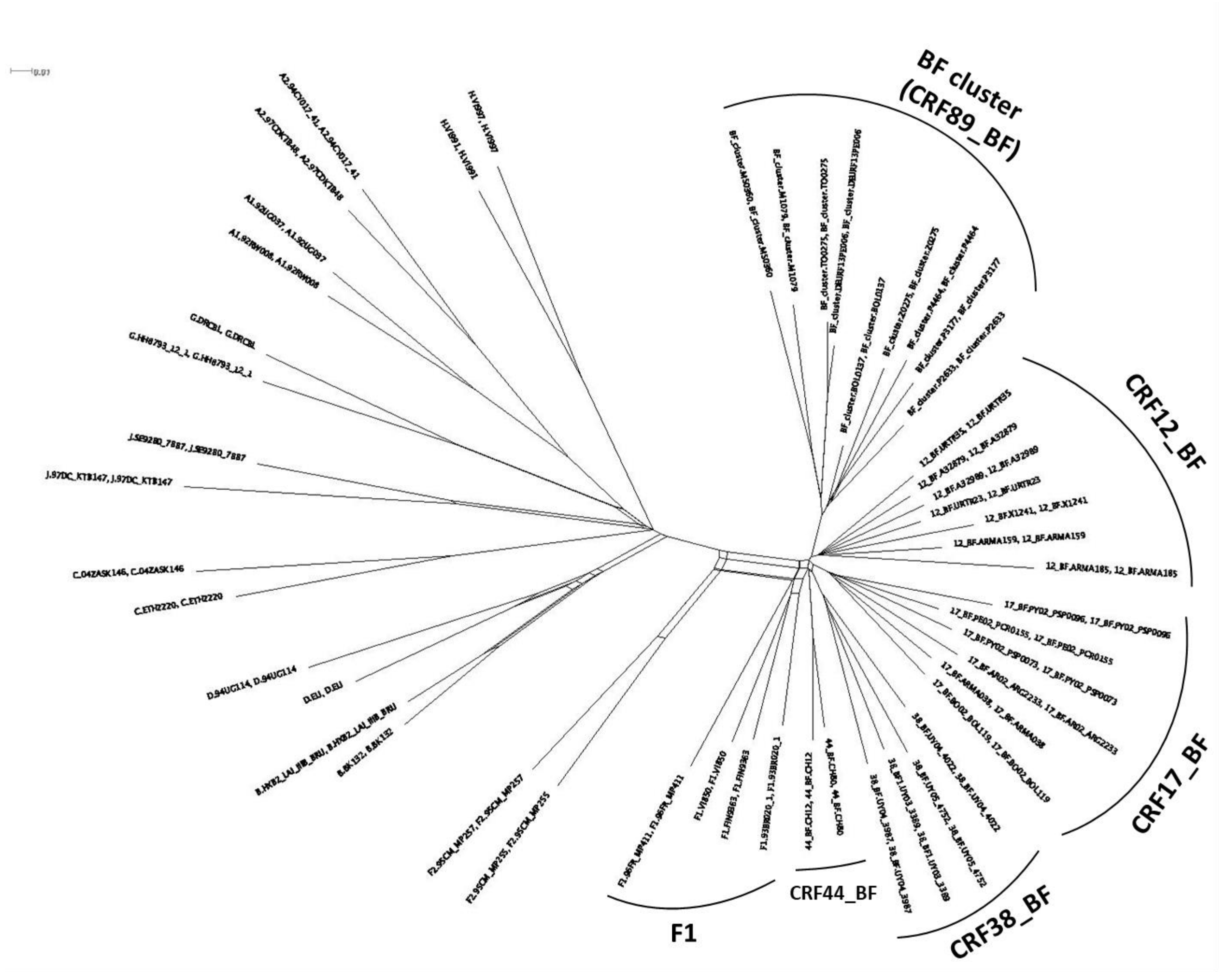

Supplementary Fig. S3. 95% confidence network of NFLG sequences of the BF cluster, analyzed together with CRF12_BF, CRF17_BF, CRF38_BF, CRF44_BF, and subtype reference sequences, constructed with Splistree4.

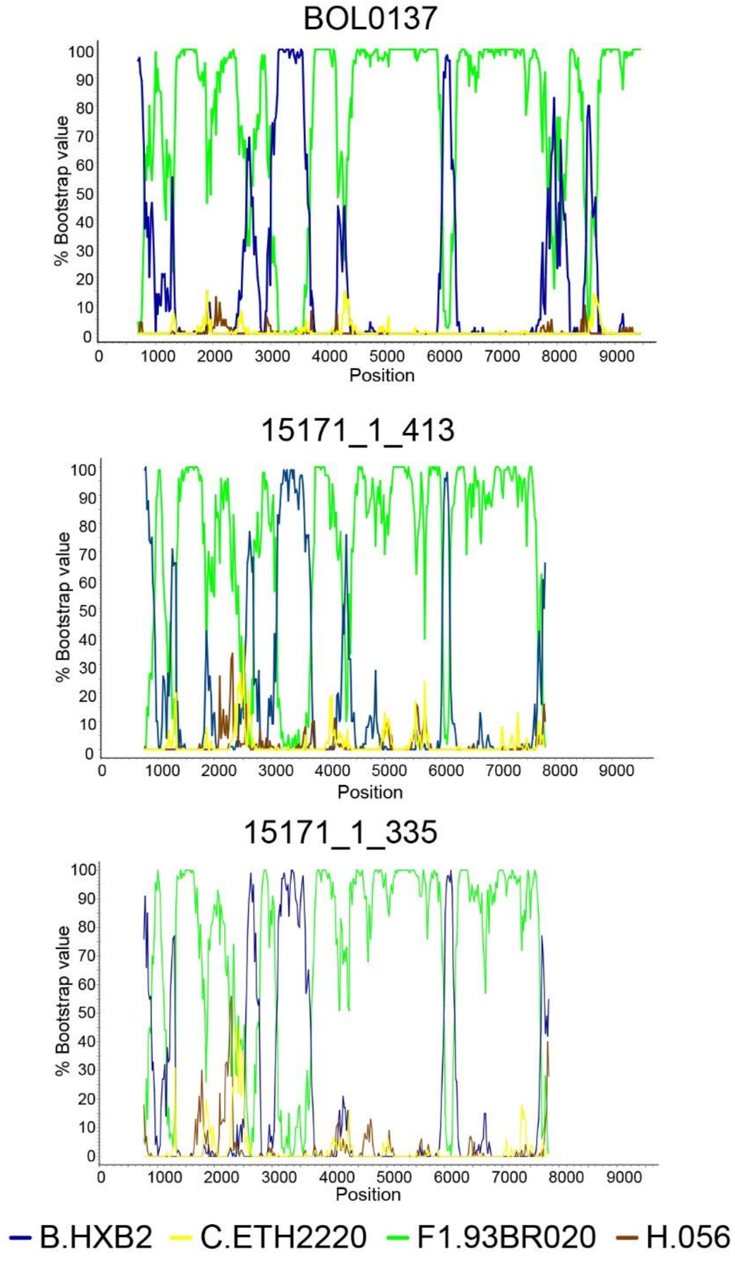

Supplementary Fig. S4. Bootscan plots of two sequences of ~7 kb of the BF cluster from UK. The horizontal axis represents the position in the HXB2 genome of the mid-point of a 250 nt window moving in 20 nt increments and the vertical axis represents bootstrap values supporting clustering with subtype reference sequences. The bootscan plot of the NFLG sequence of BOL0137 is shown on top for comparison.

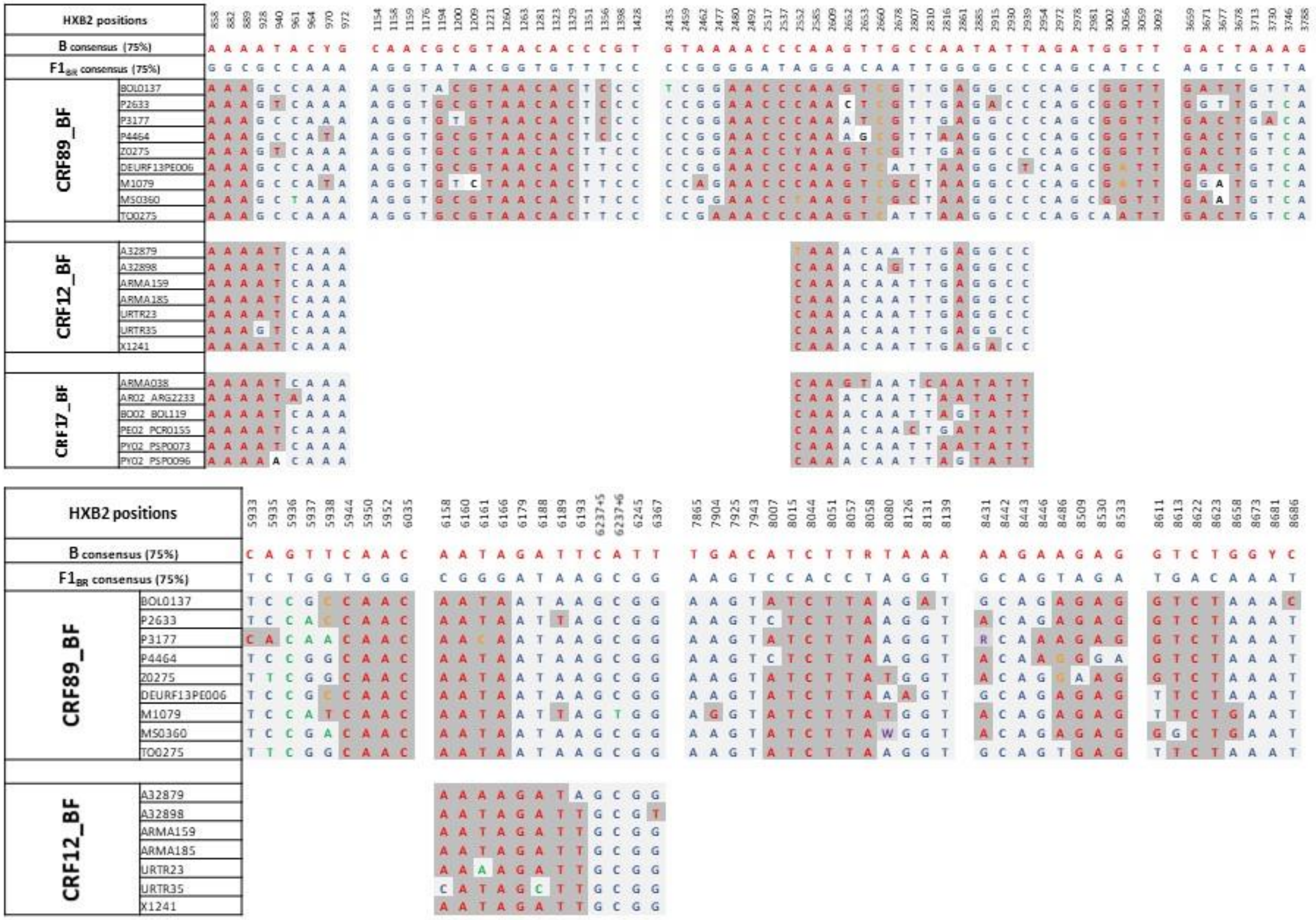

Supplementary Fig. S5. Intersubtype transitions in viruses of the BF cluster (CRF89_BF). On top of upper and lower panels, 75% consensus nts of the parental clades, subtype B and the Brazilian F1 subsubtype strain, are shown at positions (HXB2 numeration) where they differ. Nts in viruses of the BF cluster are in red or blue if they are identical to the B or F1 consensuses, respectively, and in orange or green if they coincide in nt class (purine or pyrimidine) with B or F1 consensuses, respectively, at positions in which consensuses differ in nt class. Dark and light grey backgrounds indicate coincidence with B or F1 consensuses, respectively. Mixture of nts coincident with B and F1 consensuses are in purple with intermediate gray background. Nts not coinciding with any of the subtype consensuses are in black with white background. Intersubtype transitions are shown for CRF12_BF and CRF17_BF at breakpoints where they differ from BF cluster viruses.

Supplementary Table 2. Likelihood mapping analysis of segments 1185-1339, 7975-8069, and 8466-8640.

Segment (HXB2 positions)	Virus ID	Resolved (%)	Partly resolved (%)	Unresolved (%)
1185-1339	BOL0137	85.8	4.2	10
1185-1339	P2633	85.1	0	14.9
1185-1339	P3177	86.25	3.1	10.65
1185-1339	P4464	80.35	5.75	13.9
1185-1339	Z0275	81.1	2.95	15.95
1185-1339	DEURF13PE006	80.75	6.6	12.65
1185-1339	M1079	76.45	0	23.55
1185-1339	MS0360	80.2	1.6	18.2
1185-1339	TO0275	79.35	1.65	19
7975-8069	BOL0137	87.45	3.9	8.65
7975-8069	P2633	87.85	9.15	3
7975-8069	P3177	84	4.95	11.05
7975-8069	P4464	81.15	1.25	17.6
7975-8069	Z0275	85.4	4.2	10.4
7975-8069	DEURF13PE006	75	1.2	23.4
7975-8069	M1079	81.65	0	18.35
7975-8069	MS0360	94	0	6
7975-8069	TO0275	75.2	6.95	17.85
8466-8640	BOL0137	78	10	11
8466-8640	P2633	75.8	14.1	10
8466-8640	P3177	77.2	14.5	8.65
8466-8640	P4464	81.4	5.6	13
8466-8640	Z0275	88	8.1	3.9
8466-8640	DEURF13PE006	85.3	12.1	2.6
8466-8640	M1079	88.65	8.6	2.75
8466-8640	MS0360	86.9	6.6	6.5
8466-8640	TO0275	82.15	5.85	12

Percentages correspond to fully resolved, partly resolved, and unresolved quartets, with alignments comprising each BF virus and 3 subtype B, 3 subsubtype F1, and 1 subtype C viruses (the last one used as outgroup).

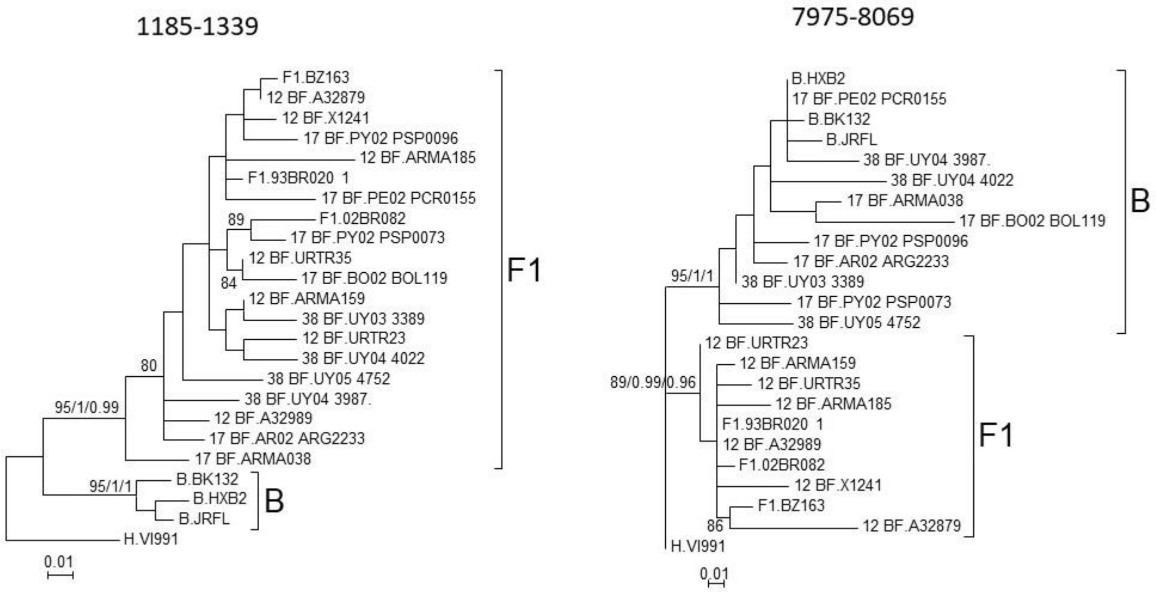

Supplementary Fig. S6. Phylogenetic trees of genome segments 1185-1339 and 7975-8069 of CRF12_BF, CRF17_BF, and CRF38_BF. Names of subtype and CRF references are preceded by the corresponding subtype or CRF. Node support values of B and F1 clades are indicated, in this order, as ultrafast bootstrap value/aLRT SH-like support/posterior probability, which were obtained with IQ-Tree, PhyML, and MrBayes programs, respectively. For the other nodes, only ultrafast bootstrap values are indicated. Only ultrafast bootstrap values ≥80% are shown.

Supplementary Table 3. Intersubtype breakpoints in BF cluster genomes detected with GARD, RDP4, and jpHMM programs.

Interclade consensus transition midpoint (interval)	909 (889-928)	1185 (1176-1194)	1340 (1329-1351)	2479 (2477-2480)	2743 (2678-2807)	2992 (2981-3002)	3696 (3678-3713)	5941 (5937-5944)	6173 (6166-6179)	7975 (7943-8007)	8069 (8058-8080)	8466 (8446-8486)	8641 (8623-8658)
**GARD**													
BOL0137	961	1169	1334	2477	2694	2972	3705	5941	6170	7967	8057	8509	8624
P2633	927	1169	1336	2477	2683	2981	3658	5941	6170	7967	8057	8449	8623
P3177	908	1185	1339	2477	2673	2981	3705	5933	6171	7973	8058	8474	8627
P4464	927	1169	1336	2481	2677	2981	3705	5935	6171	7925	8057	8574	8623
Z0275	916	1169	1261	2477	2677	2981	3706	5938	6172	7973	8079	8552	8623
DEURF13	927	1185	1339	2480	2653	2981	3706	5933	6173	7986	8057	8571	8624
M1079	880	1169	1336	2477	2652	2981	3688	5938	6171	7973	8093	8483	8671
MS0360	916	1169	1440	2477	2753	2981	3665	5929	6172	7967	8079	8505	8671
TO0275	916	1169	1336	2466	2786	3023	3705	5941	6171	ND	ND	8487	8586
**RDP4**													
BOL0137	954	1187	1337	2494	2792	2990	3673	5940	6173	7975	8070	8500	8636
P2633	899	1182	1350	2489	2792	2990	3665	5940	6173	ND	ND	8475	8672
P3177	914	1182	1346	2489	2787	2990	3693	5938	6173	7965	8068	8444	8625
P4464	899	1129	1338	2489	2787	2990	3696	5938	6173	ND	ND	8520	8634
Z0275	ND	1192	1357	2489	2787	2990	3690	5941	6173	7975	8083	8500	8635
DEURF13	ND	1178	1327	2489	2787	2994	3685	5941	6173	ND	ND	8500	8626
M1079	ND	ND	ND	2460	2883	2990	3729	5940	6173	ND	ND	8506	8660
MS0360	ND	ND	ND	2478	2703	3053	3666	5952	6169	7985	8104	8491	8660
TO0275	ND	1178	1327	2470	2672	3053	3692	5941	6173	7946	8069	8500	8653
**jpHMM**													
BOL0137	ND	1185±8	1403±46	2488±25	ND	ND	3705±24	5940±2	6173±6	ND	ND	8485±22	8648±25
P2633	940±20	1185±13	1395±39	2338±33	ND	ND	3739±59	5941±2	6173±6	ND	ND	8493±16	8670±44
P3177	909±9	1183±11	1392±33	2472±33	ND	ND	3790±19	5941±2	6173±6	ND	ND	8481±18	8655±22
P4464	848±58	1183±11	1392±33	2492±23	ND	ND	3745±63	5933±11	6173±6	ND	ND	8498±6	8676±7
Z0275	ND	1183±10	1375±22	2492±23	ND	ND	3705±23	5940±2	6173±6	ND	ND	8512±19	8649±22
DEURF13	914±23	1183±10	1375±23	2489±27	ND	ND	3746±63	5940±2	6173±6	ND	ND	8461±10	8671±36
M1079	919±29	ND	ND	2477±38	ND	ND	3648±22	5941±2	6173±6	ND	ND	8437±48	8655±25
MS0360	915±6	1183±10	1389±64	2492±24	ND	ND	3718±11	5940±3	6173±6	ND	ND	8430±56	8653±28
TO0275	ND	1183±10	1389±64	2400±77	ND	ND	3750±58	5941±3	6173±6	ND	ND	8497±5	8688±20

On top, intersubtype consensus transition intervals (using as parental references 75% consensuses subtype B and the Brazilian subsubtype F1 strain, see supplementary Fig. S5) and respective midpoints are shown. Breakpoints falling more than 50 nt outside of those intervals are in red. ND: breakpoint not detected near the corresponding interval. Shadowed areas represent subtype B segments delimited by the breakpoints detected by each program.

Supplementary Table 4. Signature amino acid residues of CRF89_BF viruses.

	Protein	p17^{gag}	p24^{gag}	p24^{gag}	p6^{gag}	GagPolTF	Protease	Tat	Tat	Rev	Vif	Vif	Vif
	Residue position	12	6	211	30	46	12	47	63	5	136	151	154
B.HXB2		E	I	L	P	A	T	Y	Q	S	Q	A	I
CRF89_BF	**BOL0137**	**Q**	**M**	**I**	**K**	**E**	**E**	**N**	**E**	**R**	**P**	**S**	**L**
	**P2633**	E	**M**	**I**	**K**	**E**	**E**	**N**	**E**	**R**	**P**	**S**	**L**
	**P3177**	**Q**	**M**	**I**	**K**	**E**	**E**	**N**	**E**	**R**	**P**	**S**	**L**
	**M1079**	**Q**	A	**I**	**K**	**E**	**E**	**N**	**E**	**R**	**P**	**S**	**L**
	**MS0360**	E	I	**I**	**K**	**E**	**E**	**N**	**E**	**R**	**P**	**S**	**L**
	**P4464**	K	**M**	**I**	**K**	**E**	**E**	**N**	**E**	**R**	**P**	**S**	**L**
	**DEURF13PE006**	**Q**	**M**	**I**	E	G	K	**N**	**E**	**R**	**P**	**S**	**L**
	**TO0275**	**Q**	**M**	**I**	E	G	K	**N**	**E**	**R**	**P**	**S**	**L**
	**Z0275**	**Q**	**M**	**I**	**K**	**E**	**E**	**N**	**E**	**R**	**P**	**S**	**L**
CRF12_BF		E$^{(71\%)}$/K$^{(29\%)}$	L	L	Q	A$^{(86\%)}$/T$^{(14\%)}$	T$^{(71\%)}$/**E**$^{(14\%)}$/I$^{(14\%)}$	Y	Q	S	Q$^{(86\%)}$/**P**$^{(14\%)}$	T	I
CRF17_BF		E$^{(67\%)}$/K$^{(17\%)}$/**Q**$^{(17\%)}$	L	L	Q$^{(83\%)}$/P$^{(17\%)}$	A	T$^{(83\%)}$/**E**$^{(17\%)}$	Y	Q$^{(50\%)}$/K$^{(33\%)}$/P$^{(17\%)}$	S	Q$^{(83\%)}$/**P**$^{(17\%)}$	T	I$^{(67\%)}$/V$^{(33\%)}$
CRF38_BF		K	L	L	Q	A$^{(75\%)}$/T$^{(25\%)}$	T$^{(75\%)}$/K$^{(25\%)}$	Y$^{(75\%)}$/H$^{(25\%)}$	Q$^{(50\%)}$/P$^{(50\%)}$	S	Q$^{(75\%)}$/**P**$^{(25\%)}$	T	I$^{(50\%)}$/**L**$^{(50\%)}$
CRF44_BF		K	L	L	Q	A	**E**$^{(33\%)}$/K$^{(33\%)}$/Q$^{(33\%)}$	Y	Q	S	**P**	T	V
F1 (BR)		K	L$^{(75\%)}$/P$^{(17\%)}$/V$^{(8\%)}$	L	Q	A$^{(83\%)}$/T$^{(17\%)}$	T$^{(75\%)}$/A$^{(17\%)}$/K$^{(8\%)}$	Y$^{(75\%)}$/H$^{(25\%)}$	Q$^{(92\%)}$/K$^{(8\%)}$	S	Q	T	I$^{(92\%)}$/A$^{(8\%)}$

Amino acid residue positions are numbered according to the proteins of the HIV-1 HXB2 isolate. Signature amino acids of CRF89_BF (found in most CRF89_BF viruses but absent or uncommon in the related CRF12_BF, CRF17_BF, CRF38_BF, and CRF44_BF, and in the parental Brazilian F1 strain) are shown in boldface.

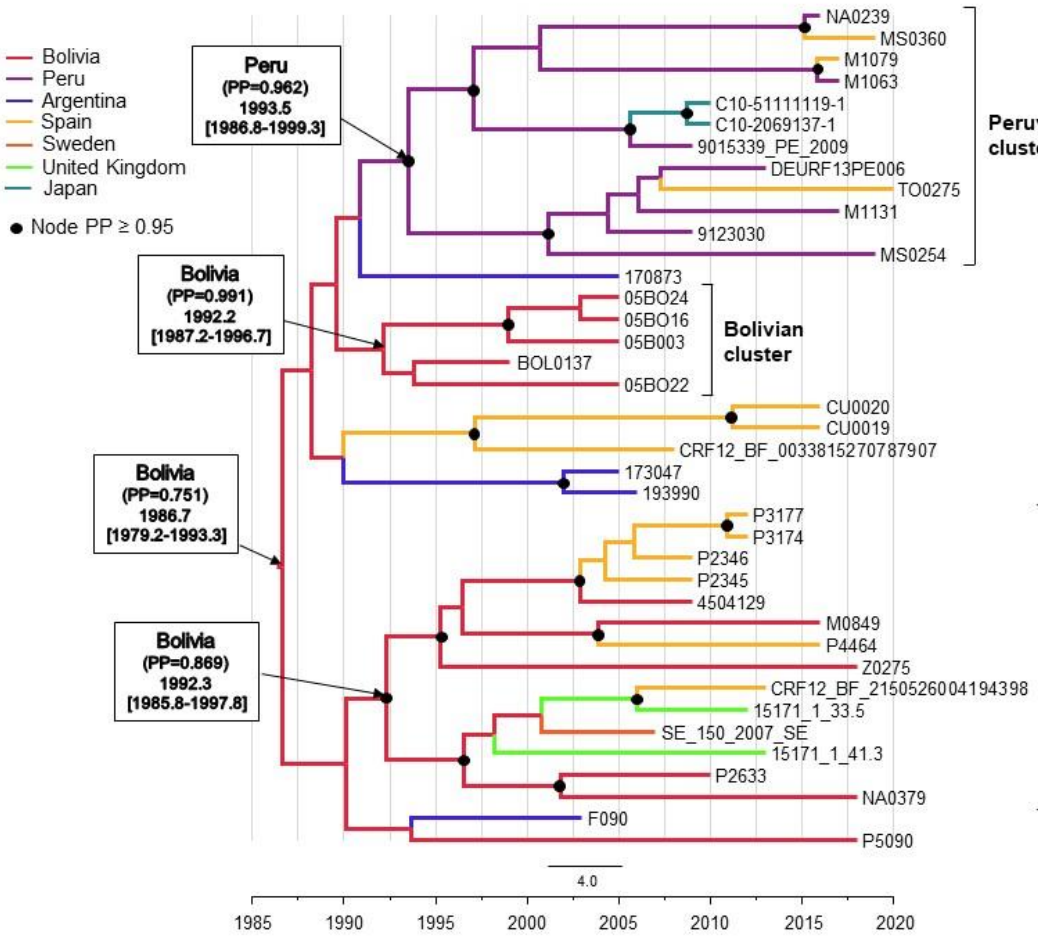

Supplementary Fig. S7. Maximum clade credibility tree of CRF89_BF Pr-RT sequences with location traits of some sequences from Bolivian and Peruvian individuals residing in Spain changed to Spain. The analysis was done with the same parameters as an earlier analysis (whose posterior tree distribution is summarized in the MCC tree shown in Fig. 6), only changing the location traits of some samples from Peruvian (M1079, MS0360) or Bolivian (P2345, P3177) individuals residing in Spain belonging to clusters of relatively recent origin from Peru or Bolivia to Spain. The rest of the figure description is as in the legend of Fig. 6.

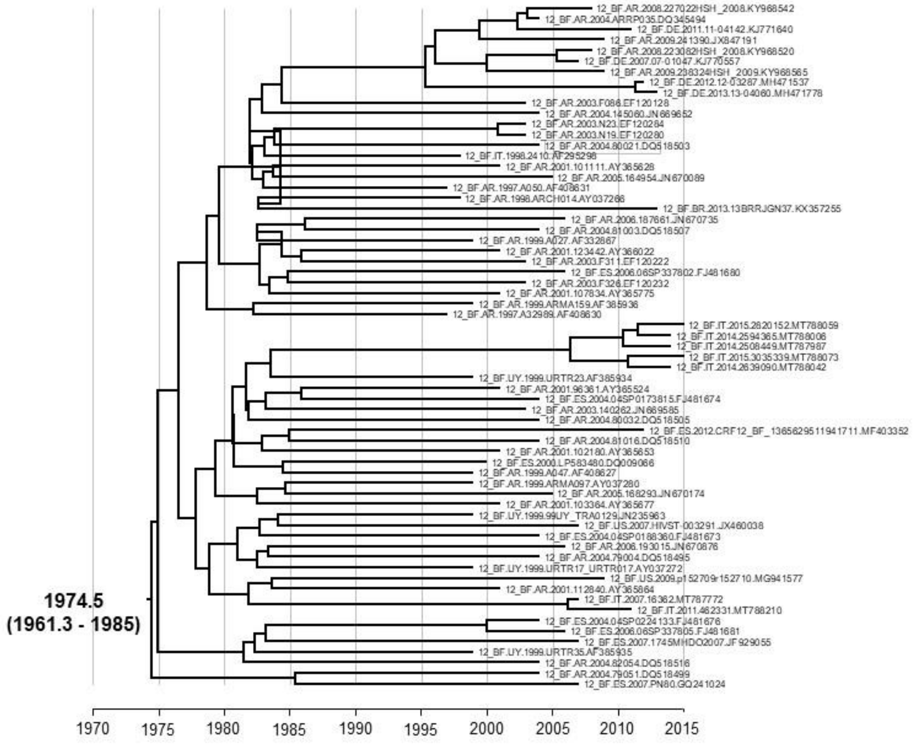

Supplementary Fig. S8. Maximum clade credibility tree of CRF12_BF Pr-RT sequences. Mean tMRCA and 95% HPD interval at the root are shown.
